# Electrocardiogram-Based Heart Age Estimation by a Deep Learning Model Provides More Information on the Incidence of Cardiovascular Disorders

**DOI:** 10.3389/fcvm.2022.754909

**Published:** 2022-02-08

**Authors:** Chiao-Hsiang Chang, Chin-Sheng Lin, Yu-Sheng Luo, Yung-Tsai Lee, Chin Lin

**Affiliations:** ^1^Division of Cardiology, Department of Internal Medicine, Tri-Service General Hospital, National Defense Medical Center, Taipei, Taiwan; ^2^Graduate Institute of Life Sciences, National Defense Medical Center, Taipei, Taiwan; ^3^Division of Cardiovascular Surgery, Cheng Hsin Rehabilitation and Medical Center, Taipei, Taiwan; ^4^School of Medicine, National Defense Medical Center, Taipei, Taiwan; ^5^School of Public Health, National Defense Medical Center, Taipei, Taiwan

**Keywords:** artificial intelligence, electrocardiography, deep learning, cardiovascular diseases, chronological age

## Abstract

**Objective:**

The biological age progression of the heart varies from person to person. We developed a deep learning model (DLM) to predict the biological age *via* ECG to explore its contribution to future cardiovascular diseases (CVDs).

**Methods:**

There were 71,741 cases ranging from 20 to 80 years old recruited from the health examination center. The development set used 32,707 cases to train the DLM for estimating the ECG-age, and 8,295 cases were used as the tuning set. The validation set included 30,469 ECGs to follow the outcomes, including all-cause mortality, cardiovascular-cause mortality, heart failure (HF), diabetes mellitus (DM), chronic kidney disease (CKD), acute myocardial infarction (AMI), stroke (STK), coronary artery disease (CAD), atrial fibrillation (AF), and hypertension (HTN). Two independent external validation sets (SaMi-Trop and CODE15) were also used to validate our DLM.

**Results:**

The mean absolute errors of chronologic age and ECG-age was 6.899 years (*r* = 0.822). The higher difference between ECG-age and chronological age was related to more comorbidities and abnormal ECG rhythm. The cases with the difference of more than 7 years had higher risk on the all-cause mortality [hazard ratio (HR): 1.61, 95% CI: 1.23–2.12], CV-cause mortality (HR: 3.49, 95% CI: 1.74–7.01), HF (HR: 2.79, 95% CI: 2.25–3.45), DM (HR: 1.70, 95% CI: 1.53–1.89), CKD (HR: 1.67, 95% CI: 1.41–1.97), AMI (HR: 1.76, 95% CI: 1.20–2.57), STK (HR: 1.65, 95% CI: 1.42–1.92), CAD (HR: 1.24, 95% CI: 1.12–1.37), AF (HR: 2.38, 95% CI: 1.86–3.04), and HTN (HR: 1.67, 95% CI: 1.51–1.85). The external validation sets also validated that an ECG-age >7 years compare to chronologic age had 3.16-fold risk (95% CI: 1.72–5.78) and 1.59-fold risk (95% CI: 1.45–1.74) on all-cause mortality in SaMi-Trop and CODE15 cohorts. The ECG-age significantly contributed additional information on heart failure, stroke, coronary artery disease, and atrial fibrillation predictions after considering all the known risk factors.

**Conclusions:**

The ECG-age estimated *via* DLM provides additional information for CVD incidence. Older ECG-age is correlated with not only on mortality but also on other CVDs compared with chronological age.

## Introduction

Cardiovascular disease (CVD) is an important leading cause of death globally, placing a heavy burden on society and families. Most guidelines for the primary prevention of CVD recommend the estimated 10-year risk as the guide to make intervention decisions (1). Pooled Cohort equations, Framingham and Systematic Coronary Risk Evaluation (SCORE) use the following variables: age, sex, cholesterol level, blood pressure, smoking, and diabetes to predict the risk of CVD (2–4). Mathematical and statistical methods converting the cardiovascular risks into heart age were developed to make it easy to understand and to reflect the biological age (3). The book, Evolutionary Biology of Aging, offered the definition of aging: a persistent decline in the age-specific fitness components of an organism due to internal physiological deterioration. However, increases in mortality with age due to chronic infections were excluded by the definition (5). Chronological age represents how long a person has been alive. Biological age is the accumulation of time, genetic, environment, lifestyle, and other unknown variables that affect aging, which means it is more interrelated with the functional status of the organism. In the past, heart age calculations based on known risk factors might not provide additional information owing to lack of unstructured data, such as image and ECG. Bone age is used to determine the skeletal maturity of children, which is useful for diagnosing short stature by comparison with chronological age (6). The success of bone age in diagnosis highlights the importance of unstructured data.

A deep learning model (DLM) is a technique used to learn useful features and provide an opportunity to speed up the process of converting unstructured data for analysis, which can also provide better accuracy in ECG interpretation (7). Previous studies have also developed a series of ECG-based DLMs on arrhythmia (8), acute myocardial infarction (9, 10), aortic dissection (11), dyskalemia (12–14), left ventricular dysfunction (15, 16), mitral regurgitation (17), aortic stenosis (18), glycemic profile (19, 20), etc. Moreover, the ECGs can even be used to predict the atrial fibrillation after a month (21). These impressive successes provided an opportunity to improve the CVD risk screening tools. A previous study used 774,783 patients to train a DLM for predicting the age of the patient, which confirmed the feasibility of age extraction from ECG (22). Furthermore, they investigated the residual between ECG-age and chronological age and found the residual is an independent predictor of all-cause mortality and cardiovascular mortality (23). Currently, many research teams had pointed out the strength of mortality risk stratification using ECG-age (24–26). However, the application potential of ECG-age was not extensively explored.

**Figure 1:**
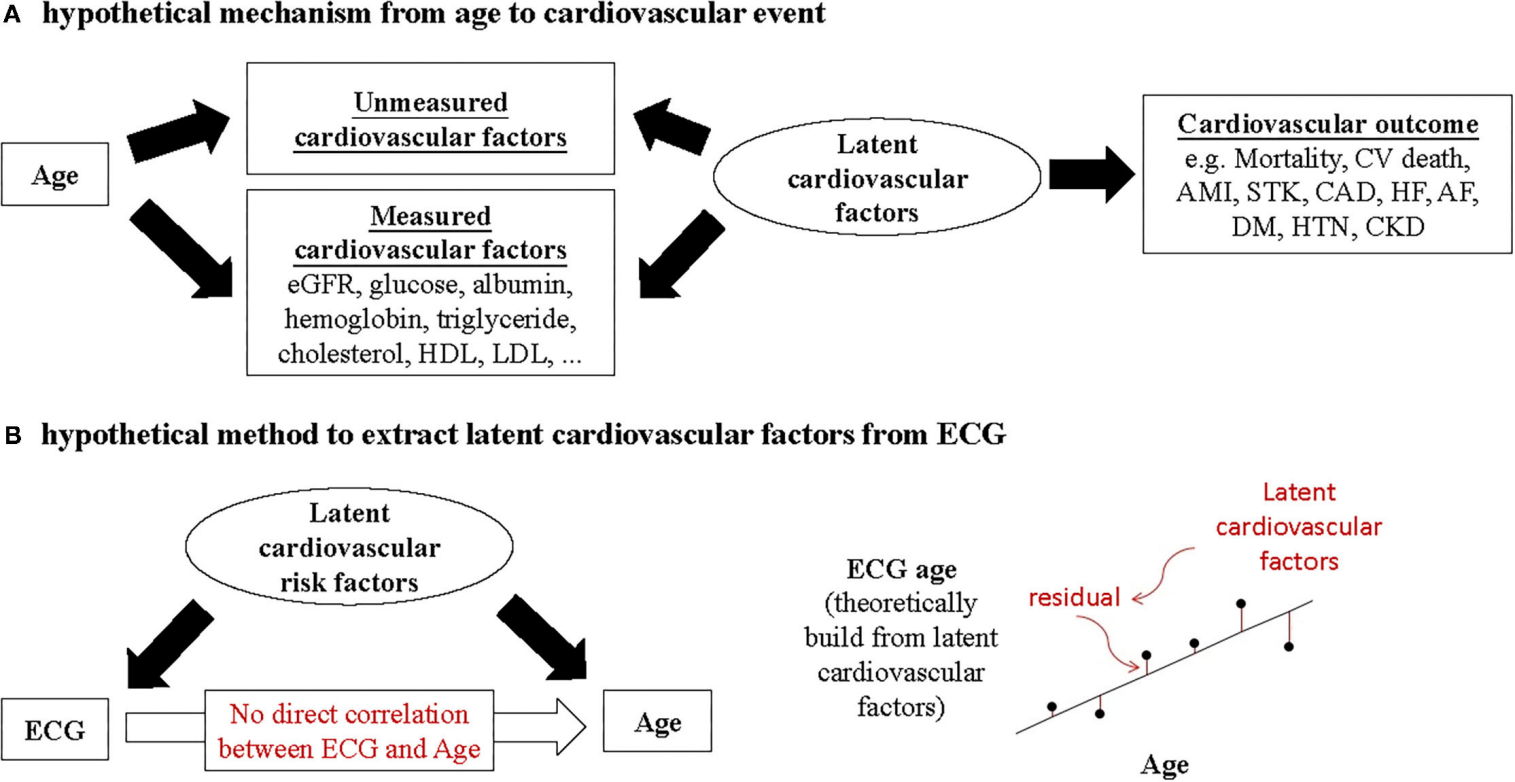
**(A)** Chronological age is not direct correlation to cardiovascular outcome. Cardiovascular outcome is correlated with the influence period of unmeasured cardiovascular factors and measured cardiovascular factors. **(B)** Under the assumption of no direct correlation between ECG and chronological age, the DLM can theoretically use the latent cardiovascular factors to generate the ECG-age for predicting chronological age. The residuals between chronological age and ECG-age may imply the information of latent cardiovascular factors. The hypothesis of why ECG-age can more accurately predict the cardiovascular outcome than the chronological age. The ECG provide more information of latent cardiovascular factors.

In our hypothesis (Figure 1), ECG-age provides more information on latent cardiovascular factors than chronologic age because ECG can reflect physiological status of the heart. The DLM can theoretically apply latent cardiovascular factors to generate the ECG-age for predicting biological age. The residual between chronological age and ECG-age may imply information of latent cardiovascular factors, which help us to promote the accuracy of CVD predictions. Here, we trained a DLM using an ECG to predict the biological age to validate the above hypothesis and to compare the correlations between chronological age, ECG-age, and the measured cardiovascular factors. Finally, we try to use the ECG-age to improve the accuracy of CVD prediction to validate the usefulness of residual between chronological age and ECG-age.

## Methods

### Data Source and Population

**Figure 2:**
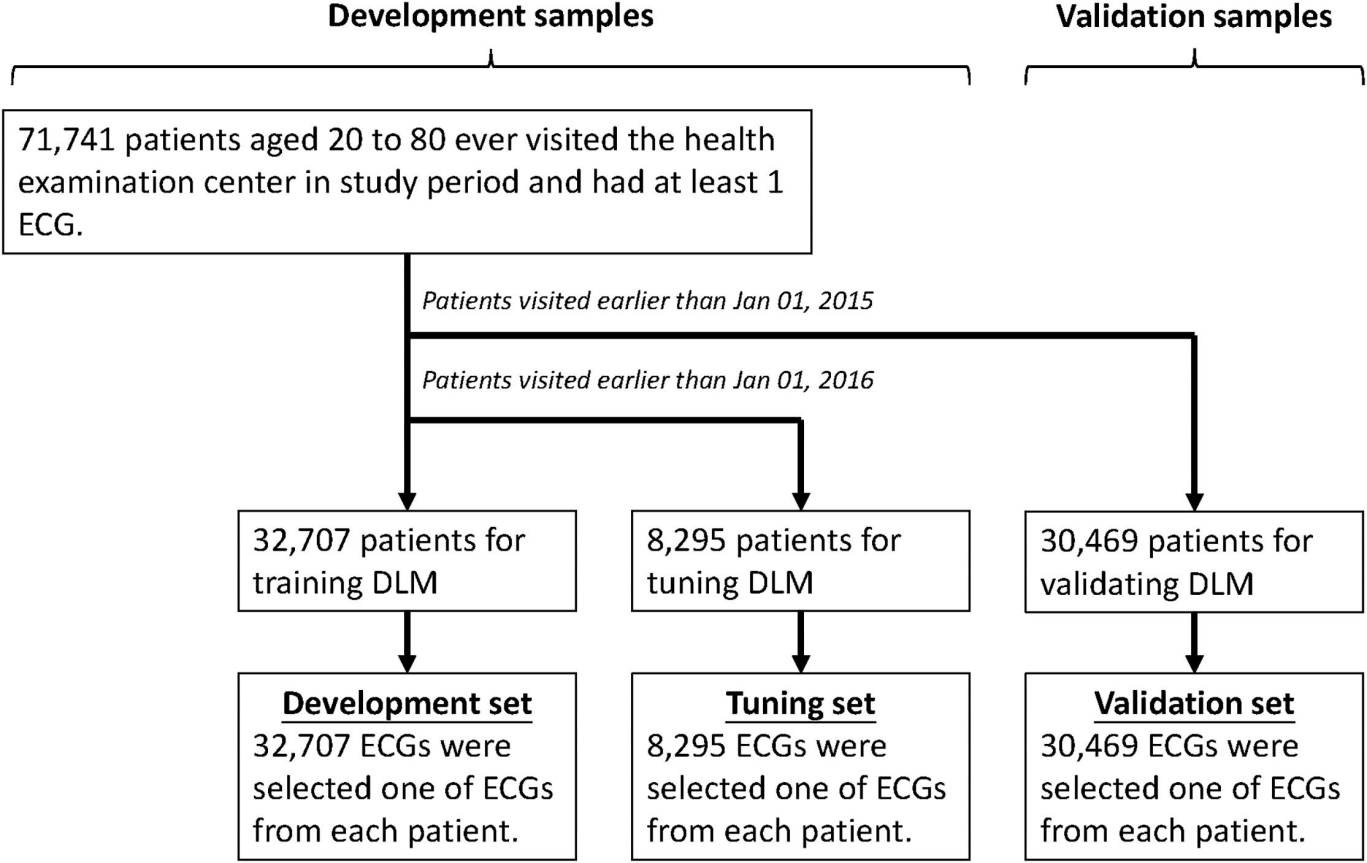
Development, tuning, and validation sets generation. Schematic of the data set creation and analysis strategy, which was devised to assure a robust and reliable data set for training, validating, and testing of the network. Once the data of patient were placed in one of the data sets, that individual's data were used only in that set, avoiding “cross-contamination” among the development, tuning, and validation sets. The details of the flowchart and how each of the data sets was used are described in the Methods.

This research was ethically approved by the institutional review board of Tri-Service General Hospital, Taipei, Taiwan (IRB No. C202005055). The electronic medical records of our hospital include digital ECG signals, and records from January 1, 2012 to December 31, 2019 were available. We only included ECGs from the health examination center to exclude the potential effect of acute diseases, and people younger than 20 years old or older than 80 years old were excluded, and Figure 2 shows the generation process of development, tuning, and validation sets. There were 71,741 first exam of ECGs and corresponding demographic characteristics in this study. We divided these ECGs into development, tuning, and validation set by date. The development set included 32,707 ECGs collected after January 1, 2016, and the corresponding ages were used to train the deep learning model, and then 8,295 ECGs collected during January 1, 2015 to December 31, 2015 were used to guide the model training as tuning set. The 30,469 ECGs collected before December 31, 2014 were used to follow the CVD-related outcomes, and cases were followed up from the date of the ECG exam to cardiovascular events or December 31, 2019, whichever came first. We also used two databases from cohort studies, the CODE-15% cohort (with 218,169 participants) and the SaMi-Trop cohorts (with 1,631 participants), to perform external validation (24).

### Observational Variables

The outcomes of interest of this study were all-cause mortality, CV-cause mortality, heart failure (HF), diabetes mellitus (DM), chronic kidney disease (CKD), acute myocardial infarction (AMI), stroke (STK), coronary artery disease (CAD), atrial fibrillation (AF), and hypertension (HTN). For the mortality data, the survival time was calculated with reference to the date of ECG. Status of the patient was defined through electronic medical records and updated by each hospital activity. New-onset cardiovascular disease is defined as when the patient was first diagnosed and documented by ICD 9 or 10 codes in our hospital electronic medical records. Moreover, data for alive visits were censored at the patient's last known hospital alive encounter to limit bias from incomplete records. The cause of death was also reviewed to distinguish a CV-cause or other reasons.

We used the corresponding International Classification of Diseases, Ninth Revision and Tenth Revision (ICD-9 and ICD-10) to define certain CVD-related outcomes. The detail codes were described as previously study (10, 13, 20, 27). For each CVD-related outcome, patients with ICD-9 or ICD-10 with corresponding diagnosis codes before the first-exam ECG collected in the physical examination center were excluded.

The ECG-age was estimated by the DLM, which is described in detail in the next section. We also collected information on chronological age, sex, body mass index (BMI), blood pressure, baseline comorbidities, and baseline biochemistry for risk evaluation and comparisons. Baseline comorbidities were extracted using ICD-9 and ICD-10 codes. Baseline biochemistry was obtained within 3 days before and after enrollment.

We used an automatic analysis system to interpret ECGs in this study. First, we divided our ECGs into normal and abnormal groups based on American Heart Association/American College of Cardiology Foundation/Heart Rhythm Society Recommendations for the Standardization and Interpretation of the Electrocardiogram, Part II: Electrocardiography Diagnostic Statement List (28). The quantitative measurements and findings within the final ECG clinical reports were extracted to identify the 31 diagnostic pattern classes and 8 continuous ECG measurements. The 8 ECG measurements included heart rate, PR interval, QRS duration, QT interval, correct QT interval, P wave axis, RS wave axis, and T wave axis. Patterns included abnormal T wave, atrial fibrillation, atrial flutter, atrial premature complex, complete AV block, complete left bundle branch block, complete right bundle branch block, first degree AV block, incomplete left bundle branch block, incomplete right bundle branch block, ischemia/infarction, junctional rhythm, left anterior fascicular block, left atrial enlargement, left axis deviation, left posterior fascicular block, left ventricular hypertrophy, low QRS voltage, pacemaker rhythm, prolonged QT interval, right atrial enlargement, right ventricular hypertrophy, second degree AV block, sinus bradycardia, sinus pause, sinus rhythm, sinus tachycardia, supraventricular tachycardia, ventricular premature complex, ventricular tachycardia, and Wolff-Parkinson-White syndrome. The 31 clinical diagnosis patterns were parsed from the structured findings statements on the basis of the key phrases that are standard within the Philips system.

### The Implementation of a Deep Learning Model

We have developed a DLM, ECG12Net, with 82-layer convolutional layers and an attention mechanism for potassium concentration estimation. The technology details, such as the model architecture, data augmentation, and model visualization, were described previously (10, 13, 27). We used the same architecture to train a new DLM for linking the ECG and the chronological age. The estimated age ranged from 20 years old to 80 years old is called ECG-age.

The ECG recordings were collected using a Philips 12-lead ECG machine (PH080A). The sampling frequency was 500 Hz, with 10 s recorded in each lead. The standard input format of ECG12Net is a length of 1,024 numeric sequences, and the original length of our 12-lead ECG signal is 5,000. In the training process, we randomly cropped a length of 1,024 sequences as input. For the inference stage, the 9 overlapping lengths of 1,024 sequences based on interval sampling were used to generate predictions and averaged as the final prediction. All parameters of the networks were trained jointly using the optimization algorithm, Adam, with standard parameters. We trained the networks with mini-batches of size 36 and used an initial learning rate of 0.001 that was decayed by a factor of 10 each time the training loss plateaued after an epoch. The only regularization method for avoiding overfitting was a weight decay of 10^−4^. Our DLM was implemented based on the software package MXNet version 1.3.0. The presented performance in the validation cohort was only evaluated once, and the estimated ECG-age in the follow-up cohort was used to predict the CVD-related outcomes. This system is configured to visualize the basis for the AI predictions using class activation mappings (CAMs) and attention mechanism (10, 11, 13, 20, 29).

### Statistical Analysis and Model Performance Assessment

We presented the characteristics of the different sets as the means and standard deviations, numbers of patients, or percentages. These values were compared using either analysis of variance or the chi-square test. The performance of the DLM was evaluated by the mean absolute errors, which were calculated in both the validation set and the tuning set. We used the scatter plot and Pearson correlation coefficient to compare the correlations between chronological age and ECG-age. Univariate and multivariable Cox proportional hazard models were used to evaluate the predictive ability of ECG-age, chronological age, and other characteristics for CVD-related outcomes, which used standardized hazard ratio (HR) and 95% confidence interval (95% CI) for comparison. We used an R package “pspline” version 1.0–18 to perform and to fit a polynomial smoothing spline of arbitrary order. For testing the proportional hazards assumption of our Cox models, we conducted the hypothesis test using a global Schoenfeld method. In principle, the Schoenfeld residuals are independent of time. A plot that shows a non-random pattern against time is evidence of violation of the proportional hazard assumption. The analyses that violated this assumption were emphasized and concluded a related conservative interpretation. A survival curve was used to visualize the people who has an older ECG-age. To evaluate the additional predictive contribution from ECG-age, we used the concordance index, also called the C-index, to present the global performance. The statistical analysis was carried out using the software environment R version 3.4.4. We used a significance level of *p* < 0.05 throughout the analysis.

## Results

**Figure 3:**
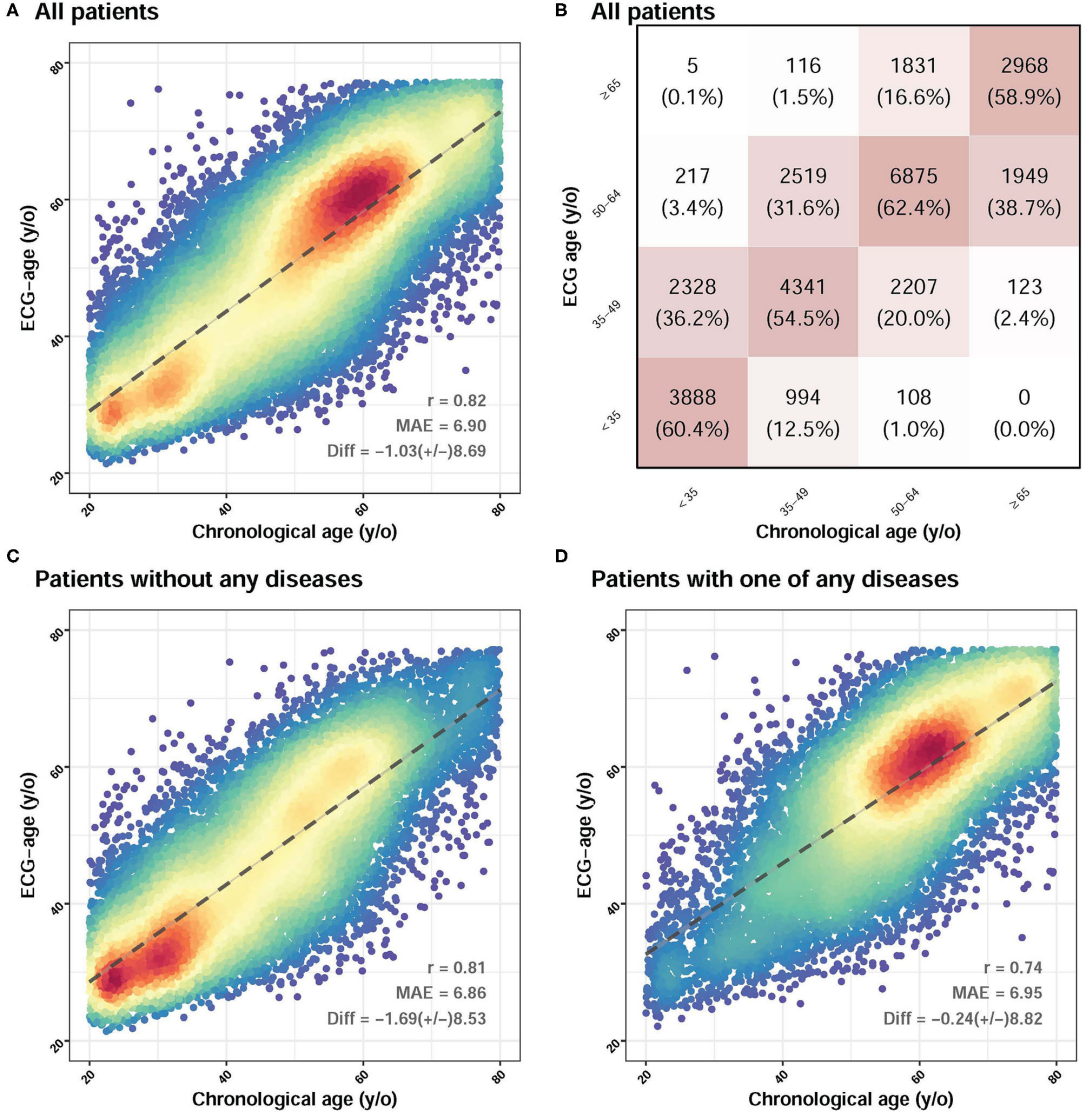
The association between chronological age and ECG-age in validation set. **(A)** Shown is the ECG age (y-axis) vs. the reported chronologic age (in years; x-axis, dotted line). The difference between chronologic age and ECG-age was 1.03 ± 8.69 years with a mean absolute error of 6.899 years. **(B)** It demonstrates a multi-group classification to the age (in years). There are 16 groups which divided by ECG age and chronologic age. The y-axis is ECG age and the x-axis is chronologic age. Each group is in terms of the percentage of patients with a specific chronologic age who had a specific corresponding ECG-age (eg, a patient from <35 y of age having a ECG age from <35 y). The squared weighted kappa value was 0.76 in this analysis. **(C)** Shown a subgroup analysis about patient without any diseases. The difference between chronologic age and ECG-age was 1.69 ± 8.53 years with a mean absolute error of 6.86 years. **(D)** Shown a subgroup analysis about patient with one of any diseases. The difference between chronologic age and ECG-age was 0.24 ± 8.82 years with a mean absolute error of 6.95 years. The MAE is highest in patient with any one of disease.

The patient characteristics in the development set, tuning set, and validation set are in Table 1. These sets showed different distributions of almost all the characteristics of the patient. As the output of the age estimation network was a continuous variable, the statistics of the mean absolute error were calculated with the overall correlation and the explained variance (R squared), which is presented in Figure 3. The difference between chronologic age and ECG-age was 1.03 ± 8.69 years with a mean absolute error of 6.899 years. The explained variance by ECG-age on chronologic age in the validation set is 66.8% (*r* = 0.822), which showed in Figure 3A. For the multi-group classification to the age groups of <35, 35 to 49, 50 to 64, and >75 and the squared weighted kappa value was 0.76 in this analysis (Figure 3B). We further evaluate the impact of different groups on MAE. We divided the patients into 2 groups. One group is patients without any diseases. Another group is patients with any one of the diseases. Figure 3C showed that the difference between chronologic age and ECG-age was 1.69 ± 8.53 years, with a mean absolute error of 6.86 years. Figure 3D showed a subgroup analysis about patients with one of any diseases. The difference between chronologic age and ECG-age was 0.24 ± 8.82 years with a mean absolute error of 6.95 years. The MAE is highest in patient with any one disease. We further explored the contribution of residuals on CVD-related outcomes.

**Table 1:** Characteristics of the patient and laboratory results in the development, tuning, and validation sets.

	**Development set (*n* = 32,707)**	**Tuning set (*n* = 8,295)**	**Validation set (*n* = 30,469)**	***p*-value**
Chronological age (years)	53.3 ± 16.9	52.8 ± 17.3	51.8 ± 16.9	<0.001
Gender (male)	18,044 (52.1%)	4,454 (50.3%)	16,726 (51.7%)	0.011
BMI (kg/m^2^)	24.6 ± 4.0	24.5 ± 4.0	24.5 ± 4.0	0.047
SBP (mmHg)	125.5 ± 19.9	124.8 ± 19.6	124.4 ± 19.5	<0.001
DBP (mmHg)	78.4 ± 12.4	77.9 ± 12.3	78.0 ± 12.3	<0.001
Disease history				
DM	6,054 (17.5%)	1,142 (12.9%)	4,008 (12.4%)	<0.001
HTN	9,509 (27.4%)	2,508 (28.3%)	10,027 (31.0%)	<0.001
HLP	9,682 (27.9%)	2,171 (24.5%)	6,935 (21.5%)	<0.001
CKD	1,312 (3.8%)	283 (3.2%)	781 (2.4%)	<0.001
AMI	362 (1.0%)	83 (0.9%)	283 (0.9%)	0.079
STK	1,913 (5.5%)	525 (5.9%)	1,829 (5.7%)	0.315
CAD	4,740 (13.7%)	1,321 (14.9%)	4,917 (15.2%)	<0.001
HF	1,147 (3.3%)	343 (3.9%)	1,518 (4.7%)	<0.001
AF	783 (2.3%)	222 (2.5%)	795 (2.5%)	0.161
COPD	3,281 (9.5%)	958 (10.8%)	3,050 (9.4%)	<0.001
Laboratory test				
GLU (mg/dL)	107.4 ± 37.7	101.9 ± 32.8	102.5 ± 33.7	<0.001
HbA1c (%)	6.0 ± 1.2	5.8 ± 1.0	5.8 ± 1.0	<0.001
TG (mg/dL)	129.5 ± 86.7	126.2 ± 81.4	127.6 ± 81.8	0.001
TC (mg/dL)	186.1 ± 39.0	187.0 ± 39.1	187.6 ± 38.3	<0.001
LDL (mg/dL)	113.2 ± 33.7	113.9 ± 33.8	115.4 ± 33.6	<0.001
HDL (mg/dL)	51.9 ± 13.7	51.7 ± 13.6	51.4 ± 13.6	<0.001
eGFR (mL/min)	91.0 ± 24.0	91.2 ± 24.0	92.3 ± 23.1	<0.001
BUN (mg/dL)	15.6 ± 9.2	15.5 ± 8.7	15.1 ± 8.4	<0.001
Na (mEq/L)	139.8 ± 2.8	140.0 ± 2.9	140.0 ± 2.9	<0.001
K (mEq/L)	4.1 ± 0.4	4.1 ± 0.4	4.0 ± 0.4	<0.001
Cl (mEq/L)	103.6 ± 3.1	103.7 ± 3.0	103.8 ± 3.1	<0.001
Ca (mg/dL)	9.4 ± 0.4	9.4 ± 0.4	9.3 ± 0.4	<0.001
Mg (mg/dL)	2.2 ± 0.2	2.2 ± 0.2	2.2 ± 0.2	0.005
AST (U/L)	22.3 ± 19.4	22.3 ± 16.3	22.2 ± 18.7	0.931
ALT (U/L)	23.3 ± 25.2	22.9 ± 25.7	23.4 ± 29.7	0.234
Alb (g/dL)	4.4 ± 0.3	4.4 ± 0.3	4.4 ± 0.4	0.009
CRP (mg/L)	0.9 ± 2.1	0.8 ± 2.0	0.9 ± 2.1	0.046
WBC (10^3^/uL)	6.5 ± 3.2	6.5 ± 2.6	6.5 ± 2.0	0.003
PLT (10^3^/uL)	245.5 ± 68.5	232.1 ± 62.6	230.9 ± 63.6	<0.001
Hb (mg/dL)	13.7 ± 1.8	13.7 ± 1.8	13.8 ± 1.8	<0.001

*BMI, body mass index; SBP, systolic blood pressure; DBP, diastolic blood pressure; DM, diabetes mellitus; HTN, hypertension; HLP, hyperlipidemia; CKD, chronic kidney disease; AMI, acute myocardial infarction; STK, stroke; CAD, coronary artery disease; HF, heart failure; AF, atrial fibrillation; COPD, chronic obstructive pulmonary disease; GLU, glucose; HbA1c, glycated hemoglobin; TG, triglyceride; TC, total cholesterol; LDL, low-density lipoprotein cholesterol; HDL, high-density lipoprotein cholesterol; eGFR, estimated glomerular filtration rate; BUN, blood urea nitrogen; Na, sodium; K, potassium; Cl, chloride; Ca, calcium; Mg, magnesium; AST, aspartate aminotransferase; ALT, alanine aminotransferase; CRP, C-reactive protein; Alb, albumin; WBC, white blood cell count; PLT, platelet; Hb, hemoglobin*.

**Figure 4:**
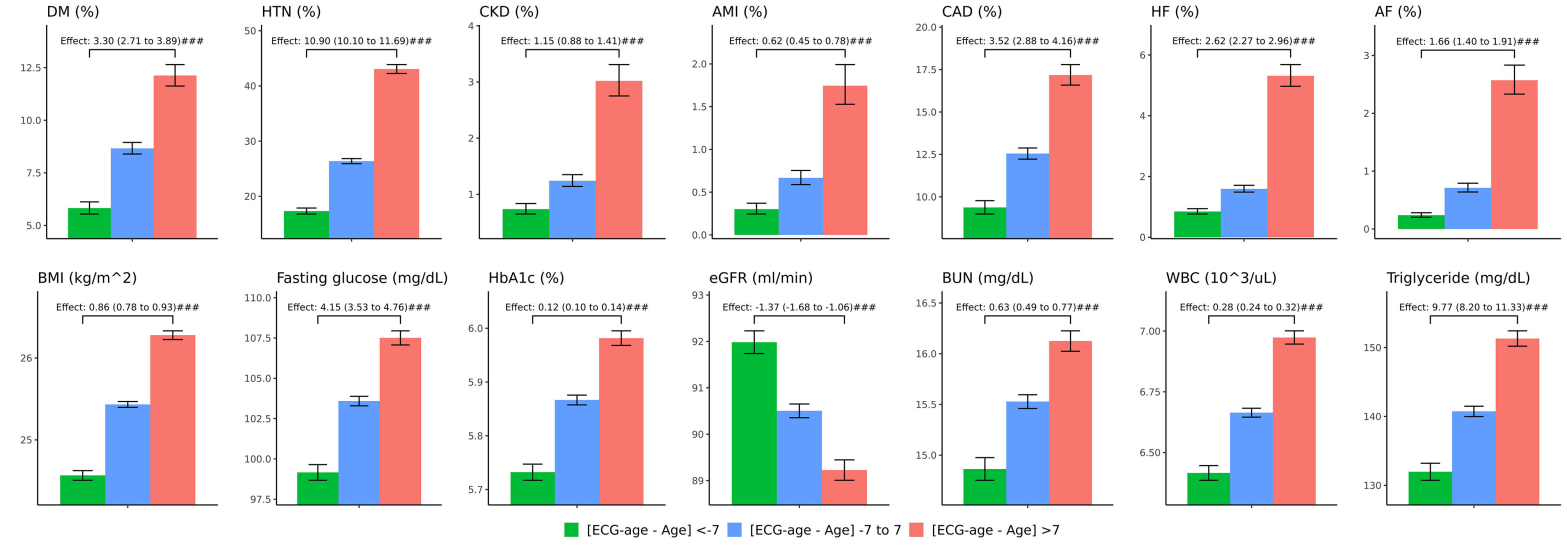
The comparison between higher ECG-age and lower ECG-age for outcomes of interest in validation set. The plots display three groups, with ECG-age more than 7 years greater than the chronological age (denoted by: ECG-age –Age >7, red columnar) those with ECG-age within a range of 7 years from their chronological age (denoted by: ECG-age –Age-7 to 7, blue columnar); and, those with ECG-age more than 7 years smaller than the chronological age (denoted by: ECG-age –Age <7, green columnar). X-axis is cumulative incidence and y axis is year. We can find red columnar is obvious with high hazard ratio on all outcomes of interest with statistics significant. Green columnar demonstrated opposite effect on all outcomes of interest.

**Figure 5:**
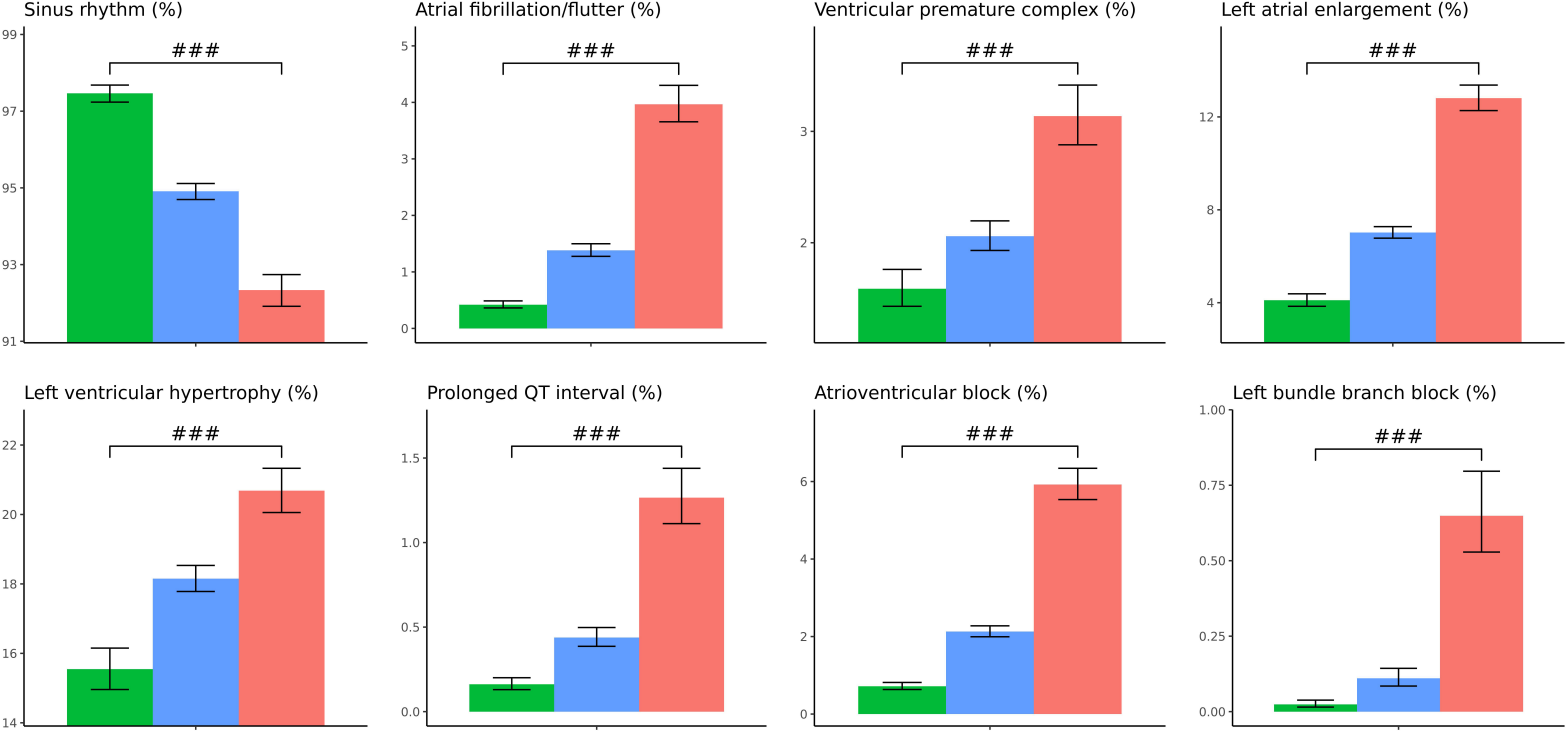
The relationship between ECG-age and ECG important features. The plots display three groups, with ECG-age more than 7 years greater than the chronological age (denoted by: ECG-age –Age >7, red columnar) those with ECG-age within a range of 7 years from their chronological age (denoted by: ECG-age –Age-7 to 7, blue columnar); and, those with ECG-age more than 7 years smaller than the chronological age (denoted by: ECG-age –Age <7, green columnar). Sinus rhythm is associated with ECG-age –Age <7, green columnar. ECG abnormal is associated with ECG-age –Age >7, red columnar. ^#^*p* < 0.05; ^*##*^*p* < 0.01; ^*###*^*p* < 0.001.

**Figure 6:**
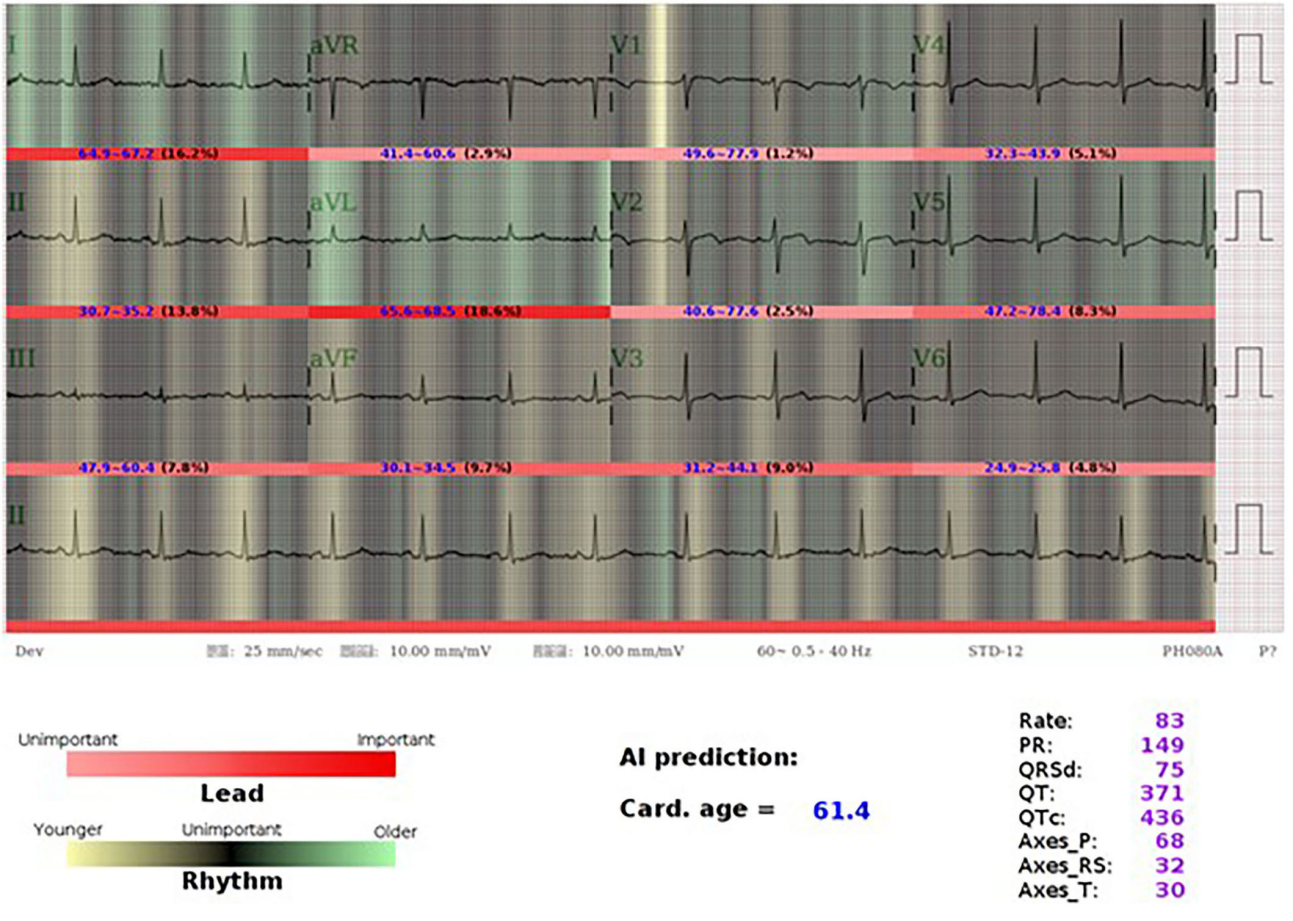
AI-ECG visualization of a 46-year old female with older ECG-age. A 46-year-old woman who had type 2 diabetes mellitus, hyperlipidemia under regular OPD follow up. Her ECG-age is 61.4 years old which is much higher than her chronological age. Using the class activation mapping and attention mechanism to explain the AI-ECG prediction, we used white-to-red gradient to indicate the importance of each lead, and the darker-to-light gradient to indicate the contribution of each position in prediction of ECG-age. Light green and yellow mean older and younger rhythms. The most important part in this case is aVL, which accounts for 18.6%, while AI considered it was an old feature with widely green color. Although it presents sinus rhythm, we can see the part emphasized by AI-ECG shows relative irregular baseline, which may be caused by a patient with muscle tremor, muscle tension, dry skin turgor, and Parkinson's disease. We considered that AI-ECG may acquire information from these tiny changes. The patient was diagnosed of coronary artery disease, single vessel disease status has post-percutaneous coronary intervention with a drug-eluting stent implanted at left circumflex artery after 3 years.

In the validation set, we further separated patients into three groups, one is high residual which means the ECG-age minus chronologic age more than 7 years, low residual which means ECG-age minus chronologic age <–7 years, and the group between high residual and low residual. In patients with discrepancy of chronologic age and ECG-age higher than 7 years, there was higher preexisting comorbidities including DM, HTN, CKD, AMI, CAD, and HF. These patients also have a higher BMI, blood urea nitrogen (BUN), fasting glucose, HbA1c, white blood cell counts (WBC), triglyceride (TG), and lower eGFR. On the other hand, those with ECG-age of 7 years less than the chronological age have a lower comorbidities and better laboratory examination. The trend test is significant between higher residual (older ECG-age) and lower residual (younger ECG-age) no matter what comorbidities or laboratory examination (Figure 4). We further analyzed the relationship between residual and all the ECG features (Supplementary Figure 1) and demonstrated the most significant ECG abnormal features related to the higher ECG-age in Figure 5. High residual is associated with more ECG abnormal features such as atrial fibrillation, left bundle branch block, atrioventricular block and ventricular premature complex, and all ECG abnormal features which revealed the statistics significance. In summary, the high residual represented more of the risk factors of CVD and might lead to a higher incidence of extensive CVD events. Figure 6 presents an ECG from a typical patient with discordant ECG-age and chronological age. In this case, the AI model detected multiple concerning features and predicted a much higher ECG-age, and, finally, the patient suffered poor outcomes despite her related low chronological age (49-year-old) initially.

**Figure 7:**
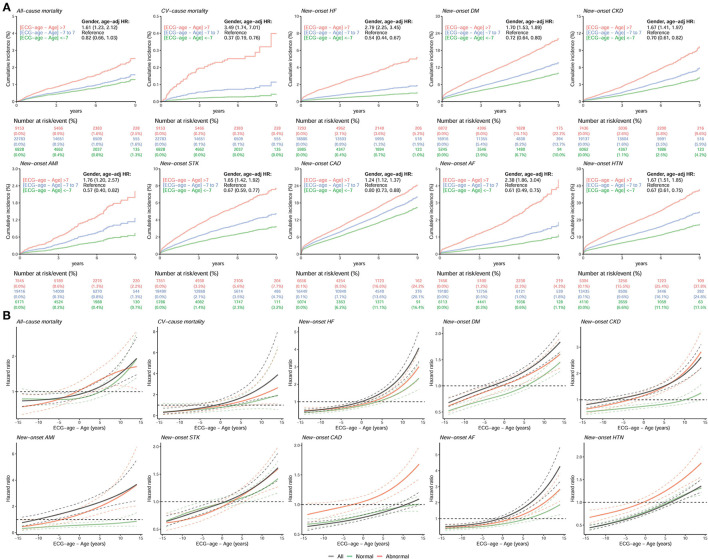
The comparison between higher ECG-age and lower ECG-age for outcomes of interest in validation set. **(A)** Long-term incidence of developing corresponding adverse event in patients at risk, stratified by the difference between chronological age and ECG-age. The table shows the at-risk population and cumulative risk for the given time intervals in each risk stratification. **(B)** Continuous association of the difference between chronological age and ECG-age on each outcome. The solid line and dashed line are the point estimation and the corresponding 95% conference interval, respectively. All hazard ratios were adjusted by gender and chronological age. “Normal” refers to the ECGs labeled as normal by the original interpreting physician at the time of ECG acquisition, “abnormal” refers to any ECGs not identified as normal. The black line, green line, and red line represent the risk curve in all ECGs, normal ECGs, and abnormal ECGs, respectively.

Figure 7A represents which new-onset CVD-related outcomes, including all-cause mortality, CV-caused mortality, HF, DM, CKD, AMI, STK, CAD, AF, and HTN, were most likely associated with a predicted ECG-age that is more than 7 years greater than the chronological age after adjustment for age and gender. We excluded the cases with corresponding histories of diseases to focus on the new-onset disease. During median follow-up years of 2.15 (interquartile range, IQR: 0.28–4.61), 2.15 (IQR: 0.28–4.61), 2.72 (IQR: 1.08–5.06), 2.55 (IQR: 0.91–4.90), 2.72 (IQR: 1.07–5.06), 2.76 (IQR: 1.10–5.12), 2.64 (IQR: 1.03–4.96), 2.47 (IQR: 0.92–4.72), 2.73 (IQR: 1.09–5.08), and 2.31 (IQR: 0.80–4.52), the at-risk patients of all-cause mortality, CV-caused mortality, new-onset HF, DM, CKD, AMI, STK, CAD, AF, and HTN initially were 38,764, 38,764, 32,164, 29,033, 32,635, 33,132, 31,636, 28,179, 32,749, and 22,849, respectively. The high residual group had an increased risk for the all-cause mortality (HR 1.61, 95%CI 1.23–2.12), CV-caused mortality (HR 3.49, 95%CI 1.74–7.01), newly onset HF (HR 2.79, 95%CI 2.25–3.45), and newly onset AF (HR 2.38, 95%CI 1.86–3.04). On the other hand, those with ECG-age of 7 years less than the chronological age have a lower cumulative incidence of new-onset CVD-related outcomes compared with predicted ECG-age range from −7 to 7 years. The low residual group had a decreased risk for CV-caused mortality (HR 0.37, 95%CI 0.19–0.76), newly onset HF (HR 0.54, 95%CI 0.44–0.67), and newly onset AF (HR 0.61, 95%CI 0.49–0.75). Not only on mortality, but also other CVD-related outcomes were demonstrated with statistical significance in a high residual group. The low residual group demonstrates opposite results compared to the high residual group. We used the spline curves showing the relationship between the new-onset CVD-related outcomes and the residual of ECG-age and chronologic age. Abnormal ECG features account for about 25% of the total in our validation set, which is 7,729 ECGs. The high residual (ECG age - age >7) is significantly increased hazard ratio of new-onset CVD-related outcomes (Figure 7B). We conducted a Global Schoenfeld test for the above Cox models (the details were shown in Supplementary Figure 2). The analyses using all the ECGs, normal ECGs, and abnormal ECGs were conducted in this assumption test. The violated analyses were new-onset AMI (*p* = 0.04091), new-onset STK (*p* = 0.001849), new-onset AF (*p* = 0.01449), and new-onset HTN (*p* < 0.00001) in all ECG, and new-onset STK (*p* = 0.01883), new-onset CAD (*p* = 0.00127), and new-onset HTN (*p* < 0.00001) in normal ECG group. In the above violated cases, some of them were contributed from sex and age, and the new-onset AMI in all ECGs (*p* = 0.0229), new-onset AF in all ECGs (*p* = 0.0016), new-onset HTN in all ECGs (*p* = 0.0004), new-onset AF in normal ECGs (*p* = 0.0359), and new-onset HTN in normal ECGs (*p* = 0.0012). These violated analyses results should be interpreted carefully. In summary, the high residual represented that the patient might lead to a higher incidence of extensive CVD events.

**Figure 8:**
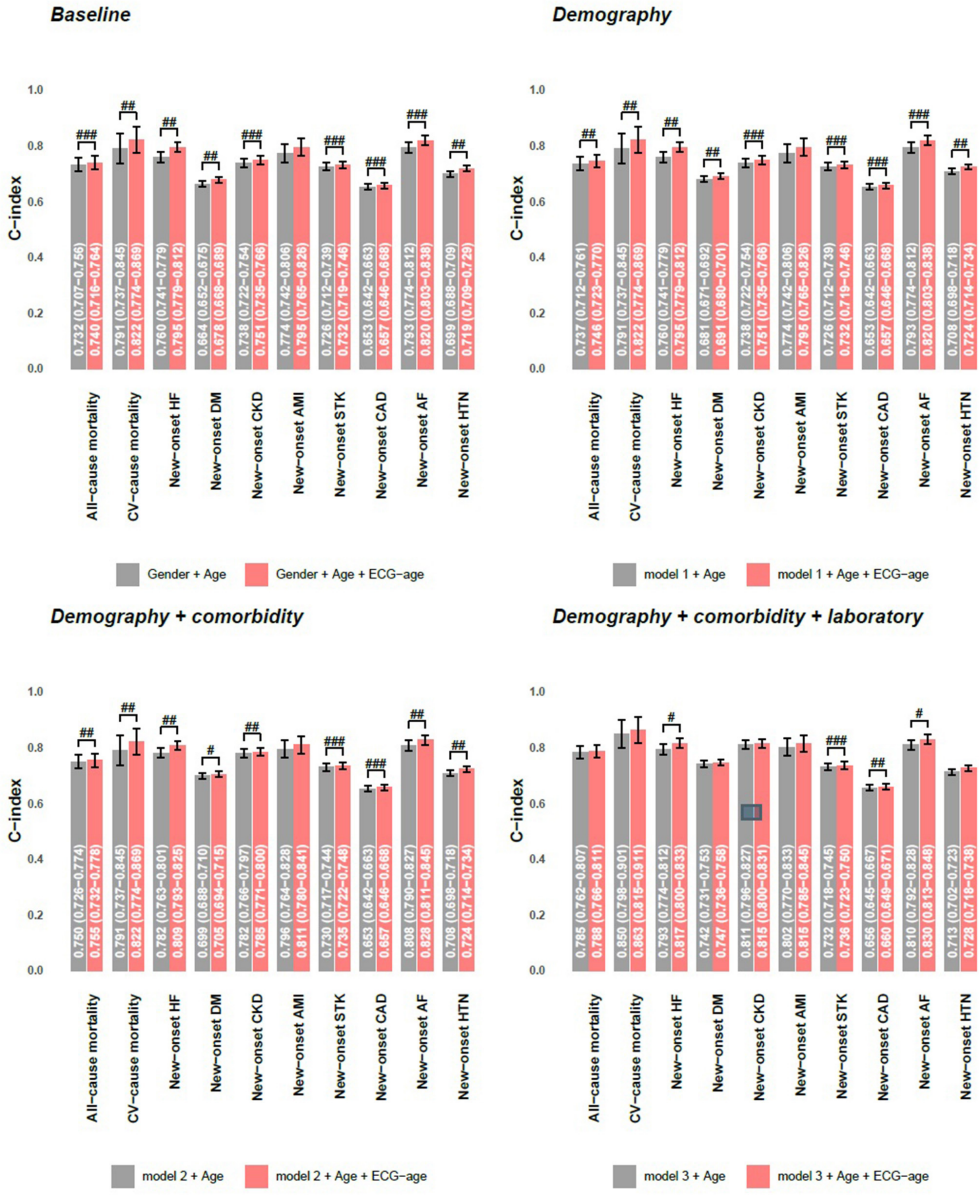
Additional contributions of ECG-age on each outcome in validation set. Cox proportional hazard model and C-index are used as the performance assessment for a series of models. The model 1 includes significant demographic data selected by stepwise program for each outcome, the model 2 includes variables in model 1 and additional significant comorbidities, and the model 3 includes variables in model 2 and additional significant laboratory tests. ^#^*p* < 0.05; ^*##*^*p* < 0.01; ^*###*^*p* < 0.001.

Cox proportional hazard model and C-index are used as the performance assessment for a series of models in Figure 8. We conducted 3 kinds of models to analyze the additional contributions of ECG-age on each outcome. Model 1 includes significant demographic data selected by the stepwise program for each outcome. Model 2 includes variables in model 1 and additional significant comorbidities, and model 3 includes variables in model 2 and additional significant laboratory tests. The more valuable variables were included, and the better C-index was conducted on each interesting outcome. In model 1 and model 2, ECG-age almost provided additional contributions on each interested outcome with statistical significance. The analysis presented here shows that the ECG-age can provide more information of the CVD-related outcomes than traditional cardiovascular risk factors. In model 3, ECG-age provided additional contributions with statistical significance on newly onset HF, newly onset STK, and newly onset AF. While not statistically significant, ECG-age demonstrated a trend to improve the C-index on each interested outcome.

**Figure 9:**
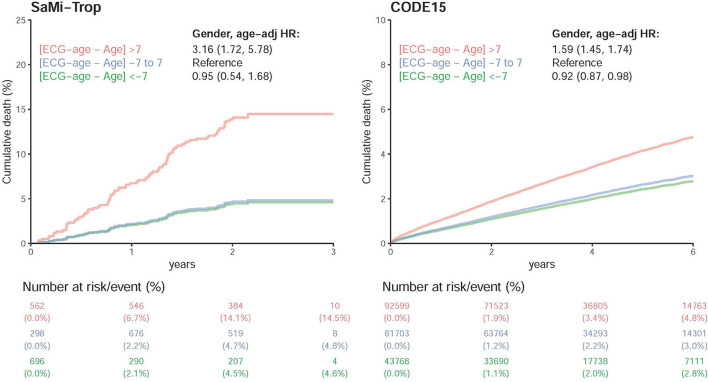
Survival analysis in SaMi-Trop and CODE15 datasets. Cox proportional model for each risk stratification on all-cause mortality in SaMi-Trop and CODE15 datasets. The table shows the at-risk population and cumulative risk for the given time intervals in each risk stratification. Both of the patients are taken into consideration: those with ECG-age more than 7 years greater than the chronological age (denoted by: “>7 years older”); those with ECG-age within a range of 7 years from their chronological age (denoted by: “±7 years”); and, those with ECG-age more than 7 years smaller than the chronological age (denoted by: “>7 years younger”).

Figure 9 shows the DLM validation in two external cohorts, the SaMi-Trop and CODE-15% (24). During median follow-up years of 2.08 (IQR: 1.98–2.23) and 3.46 (IQR: 2.12–5.22), the initial at-risk patients in SaMi-Trop and CODE-15% were 1,556, and 218,070, respectively. Whether it be the SaMi-Trop cohort or the CODE-15% cohort, the high residual revealed a high all-caused death after adjustment for age and gender. The SaMi-Trop cohort showed an HR of 3.16 (95% CI: 1.72–5.78). The CODE-15% cohort showed an HR of 1.59 (95% CI: 1.45–1.74). In low residual, it also revealed a decreased HR in all-cause mortality.

## Discussion

In this study, we trained our DLM to predict age by ECG. The mean difference between ECG-age and chronologic age was 6.899 years, which is consistent with a previous report (22). Such results indicated that the residual does not contribute to random errors. We considered that ECG-age was an independent risk factor correlated with CVD-related outcomes. In a previous study, patients with higher residual have more comorbidities such as low ejection fraction, hypertension, and coronary diseases (22). Our study evaluated the relationship between ECG-age and ECG abnormal features, and the results revealed that the high residual was the high frequency of ECG abnormal features. We further applied an ECG-age to predict CVD-related outcomes and found that patients with a high residual were significantly susceptible to an all-cause mortality, CV-caused mortality, and CVD-related outcomes after adjusting for potential confounding factors. Subgroup analysis showed the group with any one of diseases with bigger MAE than health group. In the external validation cohort, high residual was also demonstrated with an increased HR of all-cause mortality.

Traditional risk factors, such as age, sex, blood pressure, smoking, DM, and cholesterol level, have been used to predict CVD-related outcomes (2–4). Interestingly, certain ECG abnormalities have been applied to predict adverse events (30). In the previous study, they proposed 4 ECG variables [i.e., AF/AF, Q/QS, intraventricular conduction delay, and left ventricular hypertrophy (LVH) by voltage] as independent predictors for CVD-related outcomes (30). Here, we also discovered a similar correlation between AI-derived ECG-age and ECG abnormal features. The CVD guideline which published by the National Vascular Disease Prevention Alliance in Australia incorporates ECG and traditional CVD risk factors to achieve better CVD event risk prediction (31). Many studies were dedicated to obtaining more information from ECG, such as predicting serum potassium concentration (13), and the presence of AF in the future (21), or predicting mortality from a 12-lead ECG (24–26). Although current CVD risk prediction models and ECG abnormalities provide great information regarding CVD events and outcomes, they lack integration, which is not convenient for clinical use. Our study provides a practical approach model, the ECG-age, to predict not only the focus on mortality but also the CVD-related outcomes.

Under normal development, the physiological age is the same as the chronological age. Many cardiovascular risk factors can lead to an early CVD and cause morphological changes on ECG. We used a class activation mapping to see which portion is AI-focused. However, we cannot fully understand how AI can predict the age and extensive CVDs. We can only find some abnormal ECG that is correlated with old ECG age. Some pathologic tiny change was probably observed by DLM. The heuristic previous study on ECG derived the heart age connected with the Bayesian approach. They used a P-wave duration, axis of QRS and T wave, RR interval variability, and QRS root mean squared voltages to estimate heart age. This study showed a good relation between ECG-age and chronological age in the adult health. It also found highly endurance-trained athletes and patient with CVD risk factors have older ECG age (32). The QRS-T angle is also a well-established ECG feature about sudden cardiac death, ventricular arrhythmia, and an all-cause mortality. It is considered a sensitive and strong predictor of heart ventricular remodeling. The widened QRS-T angle is also correlated with age (33, 34). The AI-derived ECG-age was used to evaluate a patient who received heart transplantation. The result demonstrated that the post-transplant ECG-age correlates more closely with the donor's than the recipient's chronological age, which means that the AI-derived ECG-age can reflect more of the heart age than the chronological age (35). All of the results partially elucidate the contribution of CVD to the morphological changes of ECG. Our ECG-age may present a more precise evaluation of the heart condition than the chronological age based on the obtained ECG information.

This model exhibits an excellent predictive performance in AF and HF, for which ECG changes are related to pathological changes in the myocardium and structural changes (36, 37). In AF, the most frequent structural change of the atrial myocardium is interstitial fibrosis, which interferes with atrial conduction (38). Although these electrical signal changes are recorded by the ECG, they are difficult to observe due to subtle changes (21). With AI, these signals can be analyzed and integrated to make useful predictions (36). A previous study proposed that an AI-enabled ECG in normal sinus rhythm permits an excellent prediction of individuals with AF in the future (21). The CHF may be associated with several ECG characteristics (36), such as AF, a long PR interval, LVH, pathological Q wave, and widened QRS (37). A previous study had proposed the accurate diagnostic power for HF *via* ECG-based computer-aided detection systems (36). All of the evidence elucidates the ECG-ages on AF and CHF, and highlights the strength of our DLM in the prediction of CVD-related outcomes.

In the target and application, our system could be applied to reduce the medical costs and screen for cardiovascular disease incident, which provides beneficial effects on public health. As illustrated by the British Whitehall II cohort study, people were classified into three categories by the severity of CVD risk, who received a different frequency of screening for CVD. Such a strategy reduced the number of person-years, which were unrecognized in the high-risk group by 62%, and decreased the major CVD events by 8% in the study (39). Appropriate risk stratification equals proper distribution of resources, which means that those with the highest risk have the clearest indication for various technologies. In another field, lung age was developed *via* a pulmonary function test that compares the expected effects of aging on the pulmonary system and the presumed additional damage from tobacco smoke inhalation. Lung age is a tool to motivate people to quit smoking. A previous study reported that providing patients with their lung age was associated with higher quit rates than the general smoking cessation protocol (40). Such evidence points out the promising role of ECG-age on motivating patients to conduct a lifestyle modification. If there is more precise risk stratification, we can detect CVD earlier and provide more precise medical interventions, which achieve a better prognosis and reduce medical costs.

Some limitations of this study should be mentioned. First, this is a hospital-based retrospective study. The included samples were collected from our physical examination center, which could not represent the community-based studies. Yet, we used open access cohort databases to improve this problem, and similar results were found that high residual correlated with a higher hazard ratio of mortality. Second, there was a lack of a gold standard for the physiological age of the heart. We directly used ECG to predict chronological age, which was based on the assumption of the same aging speed of chronological age and heart age. Third, transparency, explainability, and bias are the limitations of using AI algorithms. We tried to use class activation mappings and attention mechanism to improve the explainability and provide a new figure to demonstrate it. Fourth, biological age is the accumulation of time, genetic, environment, lifestyle, and other unknown variables that affect aging. Our electronic medical records did not store this information, which may influence the biological age. This single-center study included a limited number of cases. Although the performance of our DLM has achieved the state-of-art results of previous studies, larger multicenter validations are needed. Finally, the Cox regression analyses in some outcomes of interest, such as the new-onset AF and the new-onset HTN, violated the proportional hazard assumption. Although similar risk curves were presented in all analyses that patients with the higher difference between ECG-age and chronologic age had a higher risk of CVDs, we should emphasize this limitation for future applications in these two outcomes.

In this study, we have achieved comparable accuracy to previous studies in age predictions (23–26). The ECG-age is correlated with mortality which was known by many heuristic studies (24–26). Here, we demonstrated that ECG-age was not only correlated with mortality but also extensive CVD-related outcomes. Although the future study may perform better to reduce the difference between predictions and actual value, this study still revealed a part of difference including an extensive biological meaning. Deep learning accelerates the production of new risk factor prediction models by integrating unstructured data. We have shown that DLM has the potential to provide additional prognostic information by one of the most widely used medical tests, the 12-lead ECG, which, with further study, could prove useful in a clinical context, both for risk prediction and for improving outcomes.

## Data Availability Statement

The original contributions presented in the study are included in the article/Supplementary Material, further inquiries can be directed to the corresponding author/s.

## Ethics Statement

The studies involving human participants were reviewed and approved by the Institutional Review Board of Tri-Service General Hospital, Taipei, Taiwan (IRB No. C202005055). Written informed consent for participation was not required for this study in accordance with the national legislation and the institutional requirements.

## Author Contributions

C-HC, C-SL, Y-TL, and CL conceived the idea of the study and reviewed the manuscript for final submission. Y-SL and CL screened the studies and performed data extraction. C-HC and CL drafted the manuscript. All authors were involved in the acquisition, analysis, or interpretation of data. All authors have seen and approved the manuscript and have contributed significantly to the work.

## Funding

This study was supported by funding from the Ministry of Science and Technology, Taiwan (MOST 108-2314-B-016-001 to CL, MOST 109-2314-B-016-026 to CL, MOST110-2314-B-016-010-MY3 to CL, and MOST 106-2314-B-016-038-MY3 to C-SL), the Tri-Service General Hospital, Taiwan (TSGH-C107-007-007-S02 to C-SL), the National Science and Technology Development Fund Management Association, Taiwan (MOST 108-3111-Y-016-009 and MOST 109-3111-Y-016-002 to CL), and the Cheng Hsin General Hospital, Taiwan (CHNDMC-109-19 to CL and CHNDMC-110-15 to CL).

## Conflict of Interest

The authors declare that the research was conducted in the absence of any commercial or financial relationships that could be construed as a potential conflict of interest.

## Publisher's Note

All claims expressed in this article are solely those of the authors and do not necessarily represent those of their affiliated organizations, or those of the publisher, the editors and the reviewers. Any product that may be evaluated in this article, or claim that may be made by its manufacturer, is not guaranteed or endorsed by the publisher.

## Abbreviations

CVD: cardiovascular disease
ECG: Electrocardiography
DLM: deep learning model
AMI: acute myocardial infarction
CAD: coronary artery disease
CHF: congestive heart failure
AF: atrial fibrillation
DM: diabetes mellitus
HTN: hypertension
CKD: chronic kidney disease
AI: artificial intelligence
SCORE: Systematic COronary Risk Evaluation
ECG-age: ECG-based heart age
BMI: body mass index
HR: hazard ratio
CI: conference interval
ROC: receiver operating characteristic
AUC: area under curve
eGFR: estimated glomerular filtration rate
TG: triglycerides
COPD: chronic obstructive pulmonary disease
K: potassium
Na: sodium
Cl: chloride
Ca: calcium
Alb: albumin
GLU: glucose
HbA1c: glycated hemoglobin
BUN: blood urea nitrogen
Cr: creatinine
WBC: white blood cell count
PLT: platelet
Hb: hemoglobin
AST: aspartate aminotransferase
ALT: alanine aminotransferase
TG: triglyceride
TC: total cholesterol
LDL: low-density lipoprotein cholesterol
HDL: high-density lipoprotein cholesterol.
