# Supplementary material for: Electrocardiogram-Based Heart Age Estimation by a Deep Learning Model Provides More Information on the Incidence of Cardiovascular Disorders
Source: Front Cardiovasc Med. 2022 Feb 8;9:754909. doi: 10.3389/fcvm.2022.754909 (PMC8860826; doi:10.3389/fcvm.2022.754909)
Supplement: Supplementary file 1 [file Data_Sheet_1.docx]

**Electrocardiogram-based heart age estimation by a deep learning model provides more information on the incidence of cardiovascular disorders**

**Supplementary Materials**

**Supplementary Figure 1 | The relationship between ECG-age and ECG all features.**

**Supplementary Figure 2 | Tests of proportional hazards assumption of Cox models.**


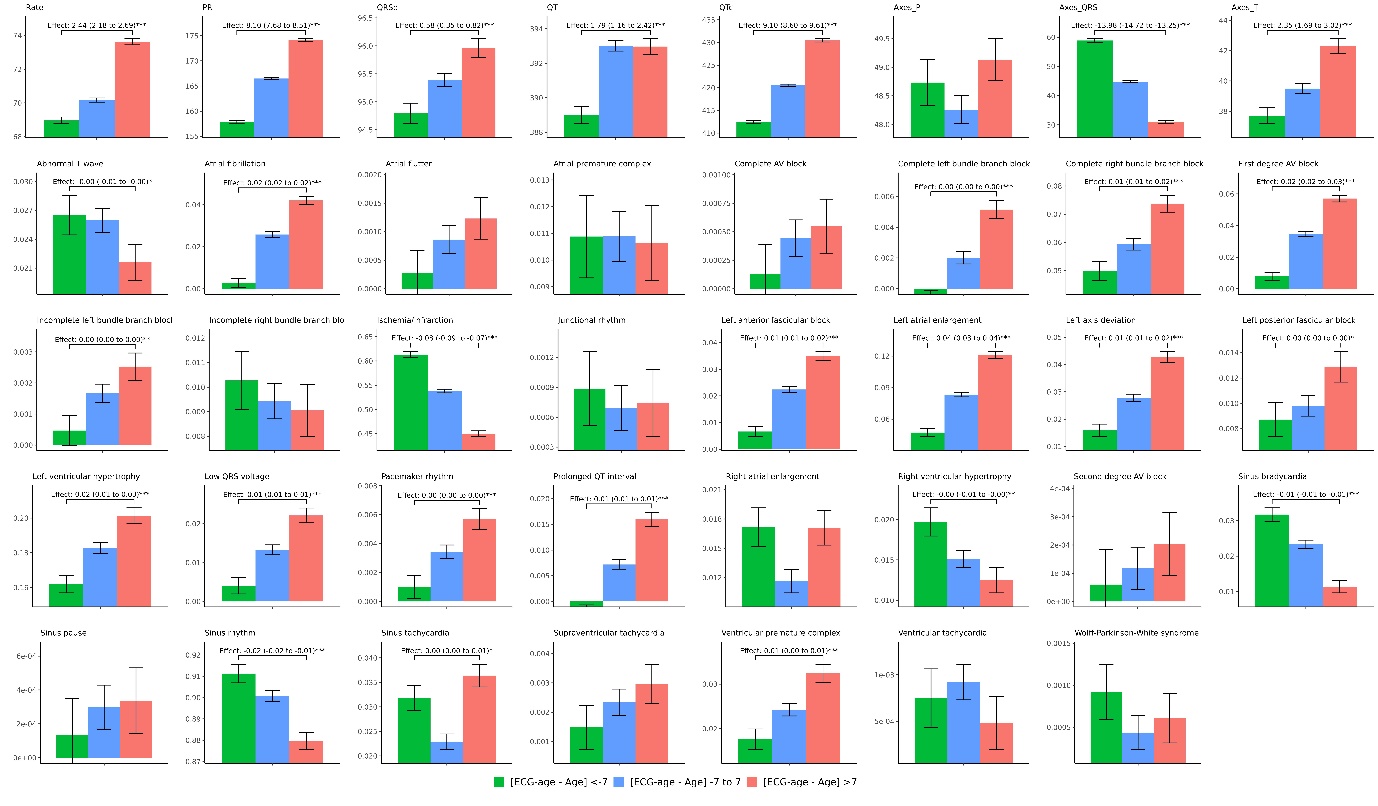


**Supplementary Figure 1 | The relationship between ECG-age and all ECG features.**

The plots display three group which ECG-age more than 7 years greater than the chronological age (denoted by: ECG-age –Age >7, red bar) those with ECG-age within a range of 7 years from their chronological age (denoted by: ECG-age –Age-7 to 7, blue bar); and, those with ECG-age more than 7 years smaller than the chronological age (denoted by: ECG-age –Age <7, green bar). Abbreviations: *, p < 0.05; **, p < 0.01; ***, p < 0.001.


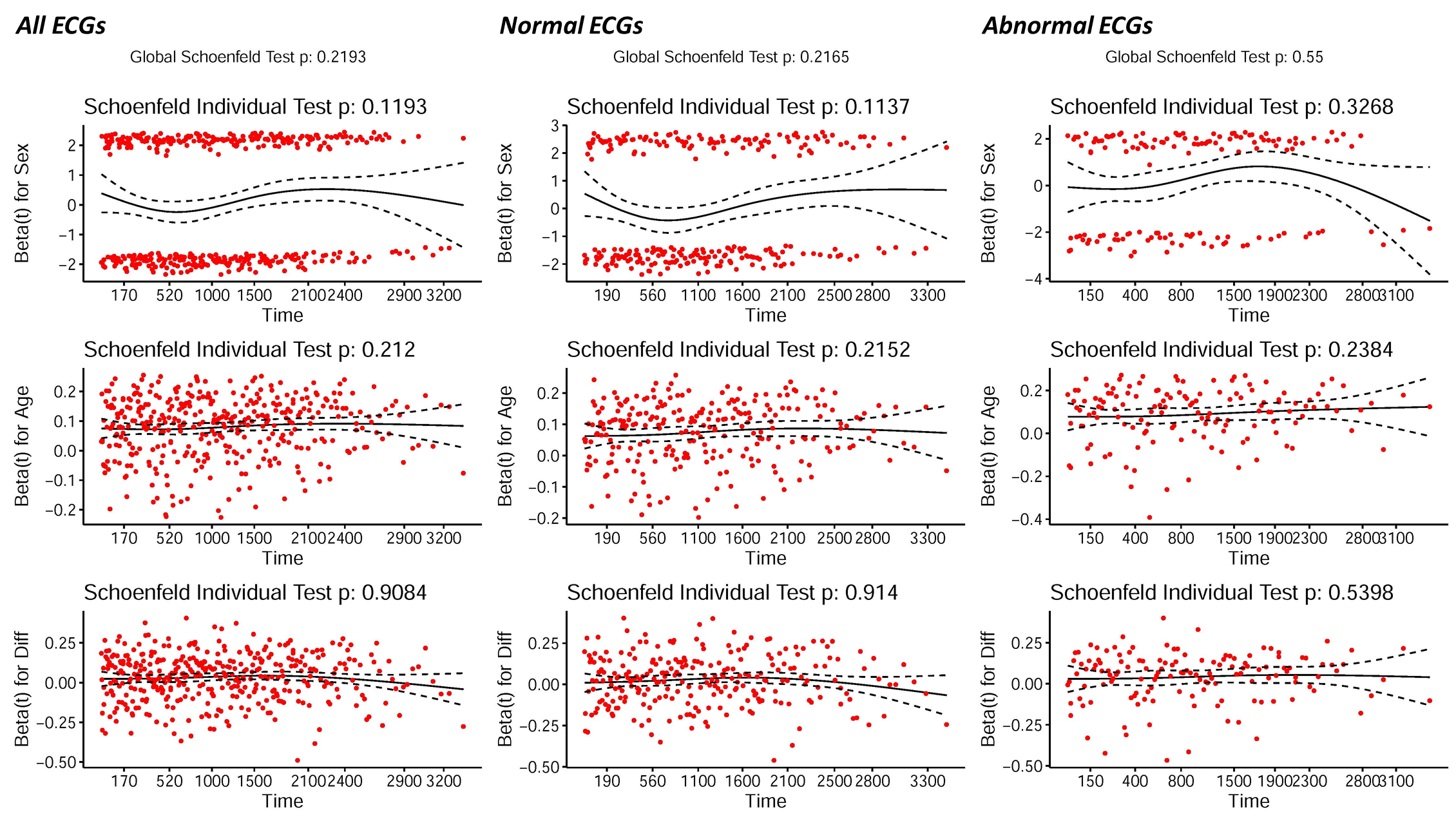


**Supplementary Figure 2-1 | Tests of proportional hazards assumption of Cox models (all-cause mortality).**

We used Global schoenfeld method to test our proportional hazards assumption of Cox models. We have three groups which is All ECGs, normal ECGs and abnormal ECGs. Each category divided into three group which is sex, age and difference (the difference between EC-age and chronologic age).


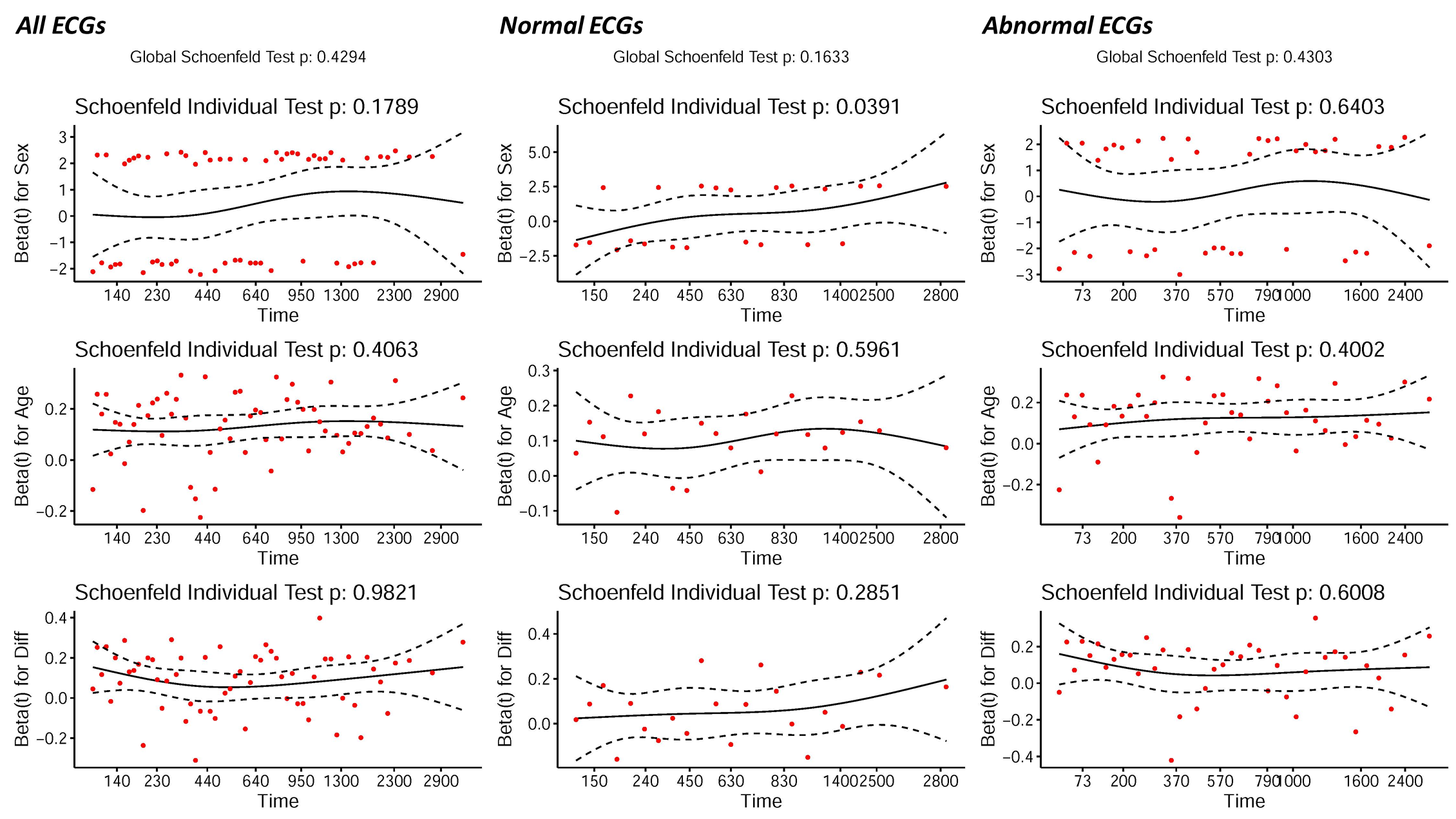


**Supplementary Figure 2-2 | Tests of proportional hazards assumption of Cox models (CV-cause mortality).**

We used Global schoenfeld method to test our proportional hazards assumption of Cox models. We have three groups which is All ECGs, normal ECGs and abnormal ECGs. Each category divided into three group which is sex, age and difference (the difference between EC-age and chronologic age).


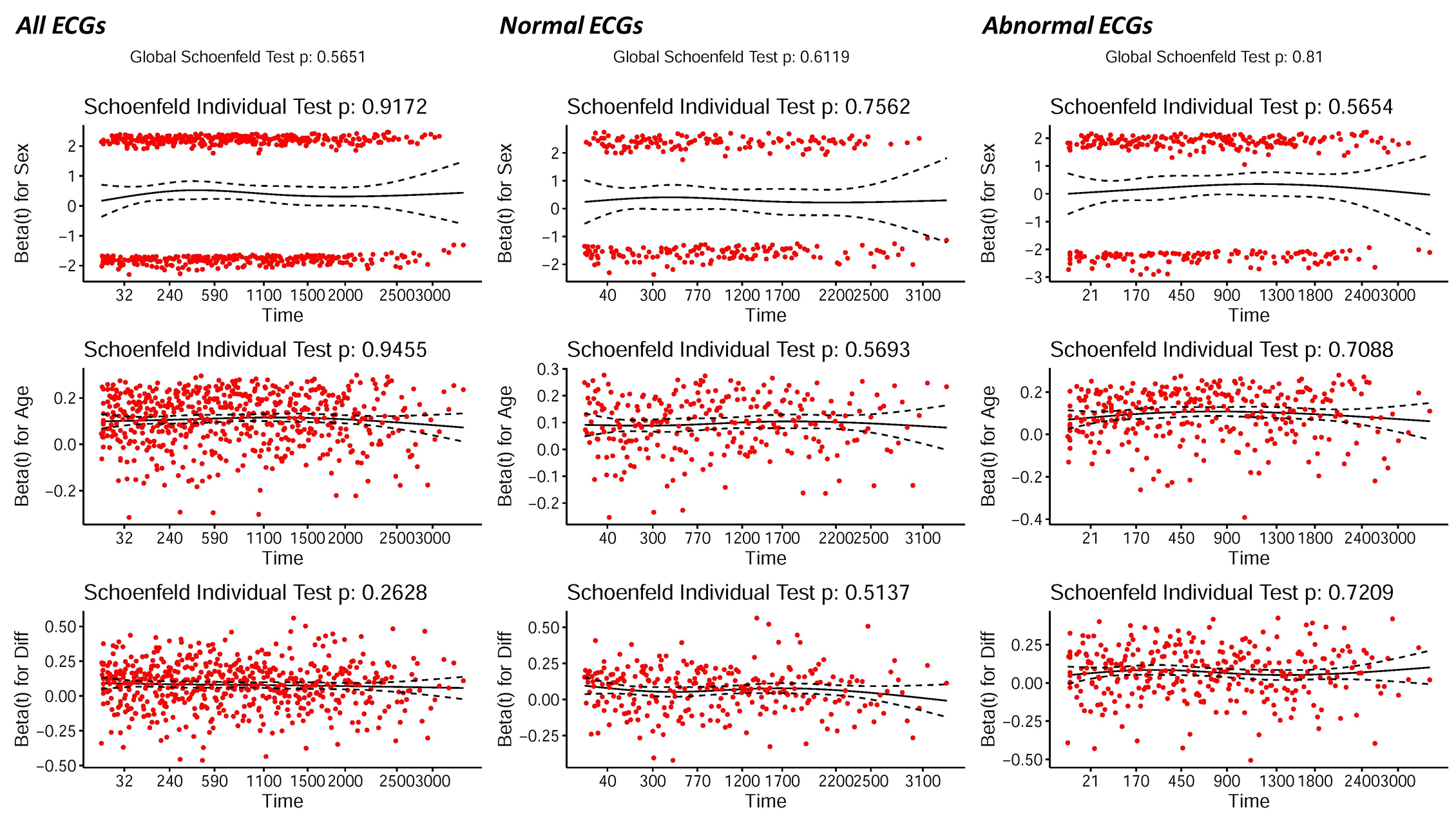


**Supplementary Figure 2-3 | Tests of proportional hazards assumption of Cox models (new-onset HF).**

We used Global schoenfeld method to test our proportional hazards assumption of Cox models. We have three groups which is All ECGs, normal ECGs and abnormal ECGs. Each category divided into three group which is sex, age and difference (the difference between EC-age and chronologic age).


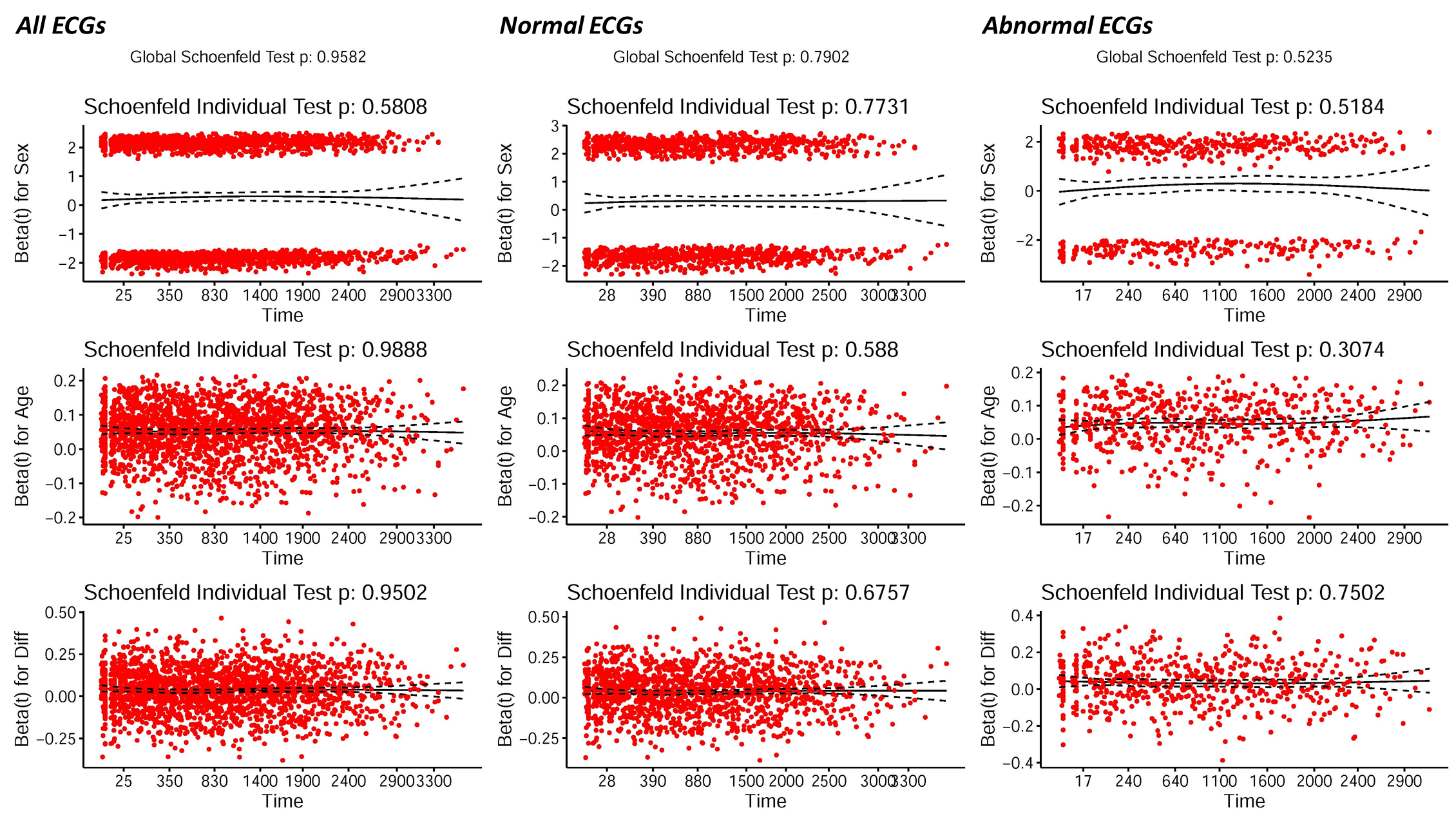


**Supplementary Figure 2-4 | Tests of proportional hazards assumption of Cox models (new-onset DM).**

We used Global schoenfeld method to test our proportional hazards assumption of Cox models. We have three groups which is All ECGs, normal ECGs and abnormal ECGs. Each category divided into three group which is sex, age and difference (the difference between EC-age and chronologic age).


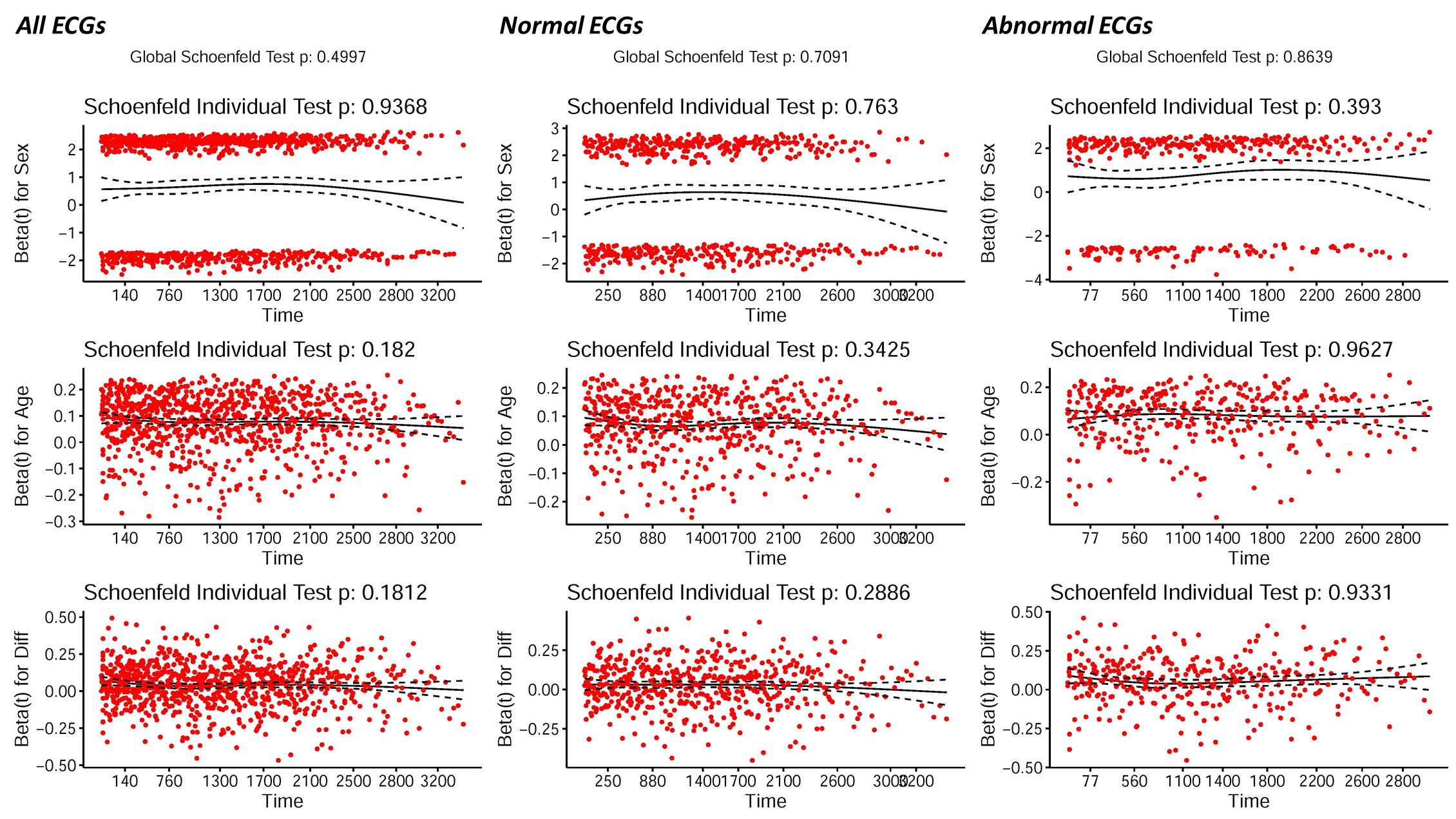


**Supplementary Figure 2-5 | Tests of proportional hazards assumption of Cox models (new-onset CKD).**

We used Global schoenfeld method to test our proportional hazards assumption of Cox models. We have three groups which is All ECGs, normal ECGs and abnormal ECGs. Each category divided into three group which is sex, age and difference (the difference between EC-age and chronologic age).


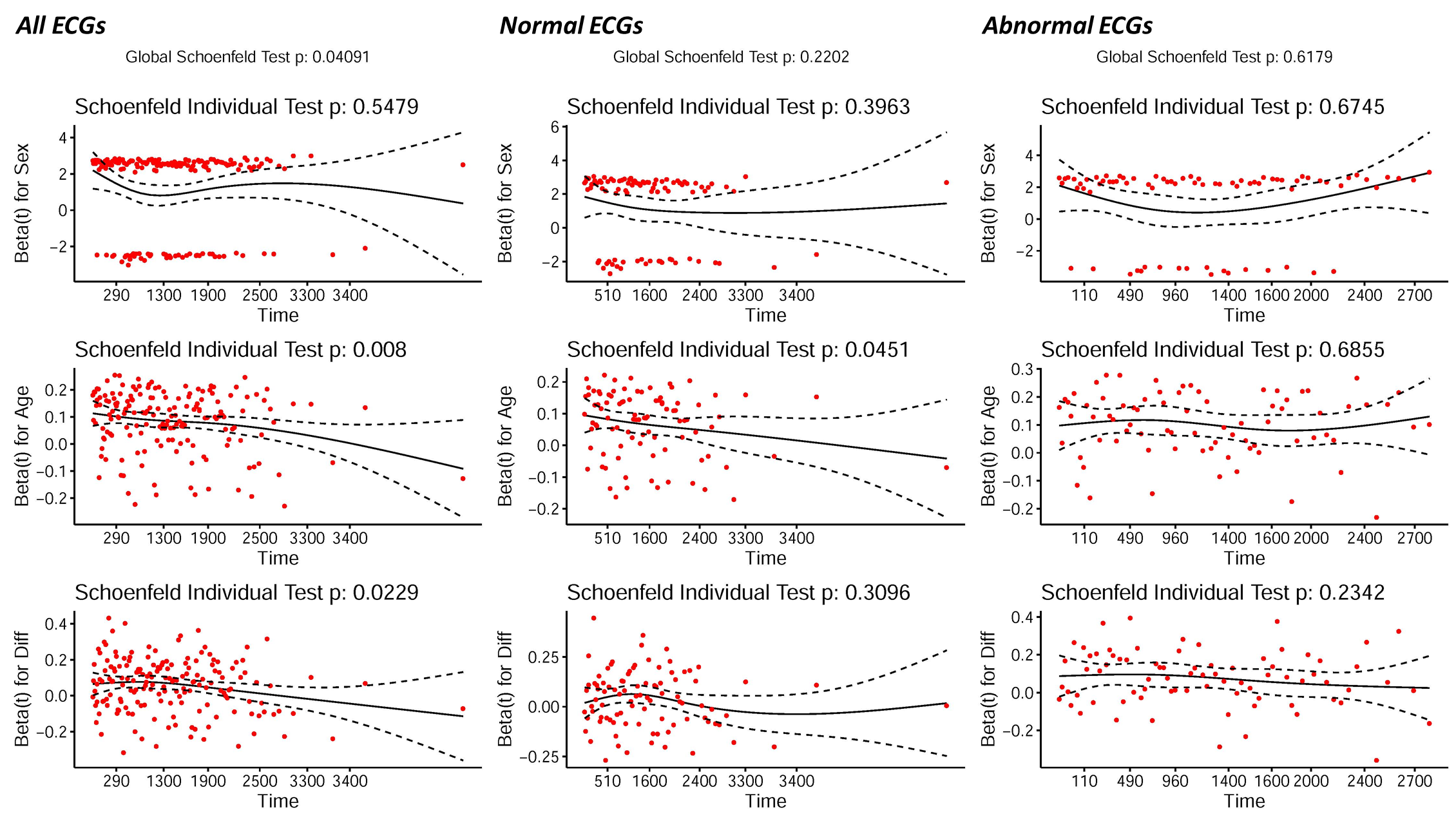


**Supplementary Figure 2-6 | Tests of proportional hazards assumption of Cox models (new-onset AMI).**

We used Global schoenfeld method to test our proportional hazards assumption of Cox models. We have three groups which is All ECGs, normal ECGs and abnormal ECGs. Each category divided into three group which is sex, age and difference (the difference between EC-age and chronologic age).


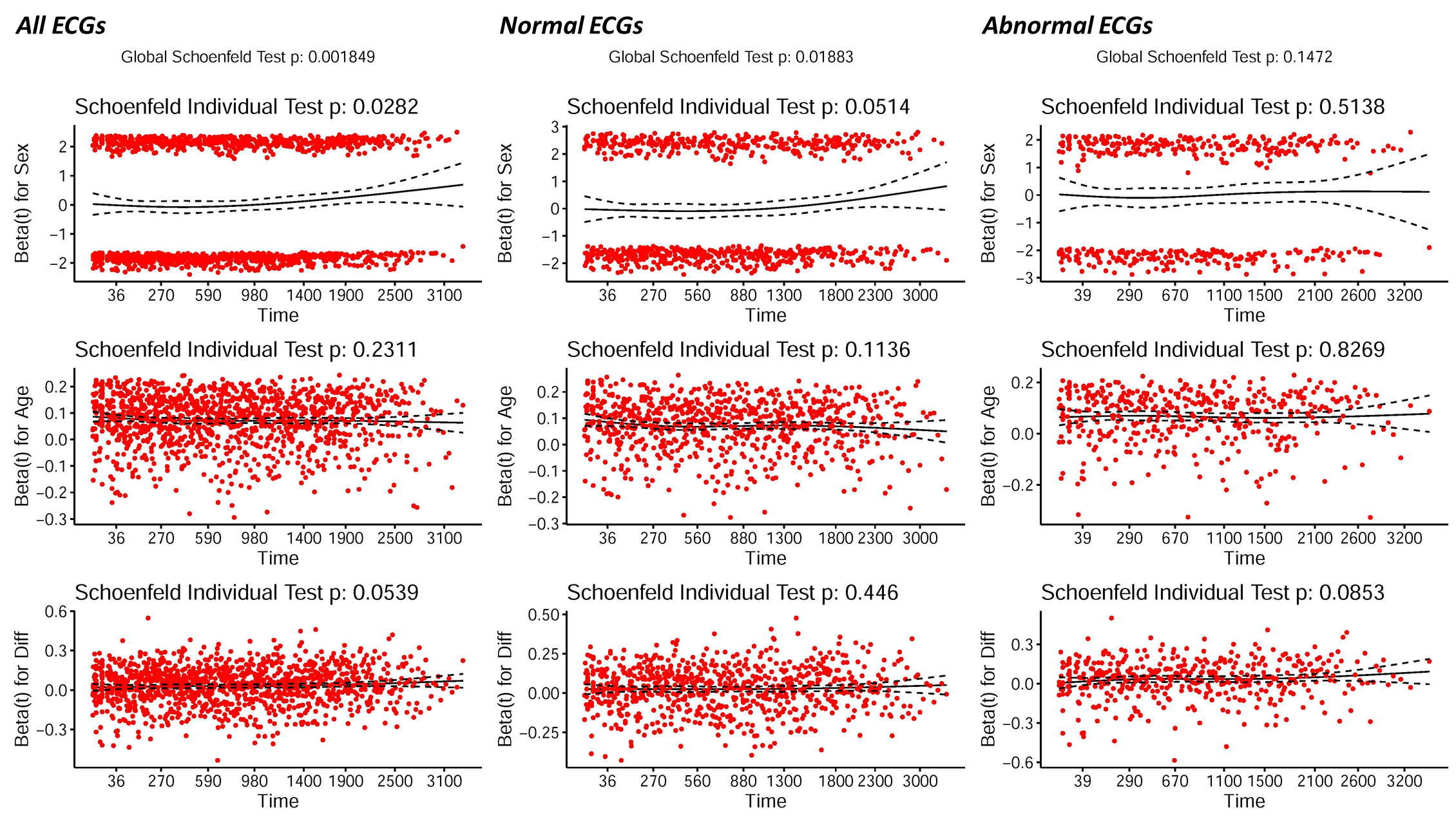


**Supplementary Figure 2-7 | Tests of proportional hazards assumption of Cox models (new-onset STK).**

We used Global schoenfeld method to test our proportional hazards assumption of Cox models. We have three groups which is All ECGs, normal ECGs and abnormal ECGs. Each category divided into three group which is sex, age and difference (the difference between EC-age and chronologic age).


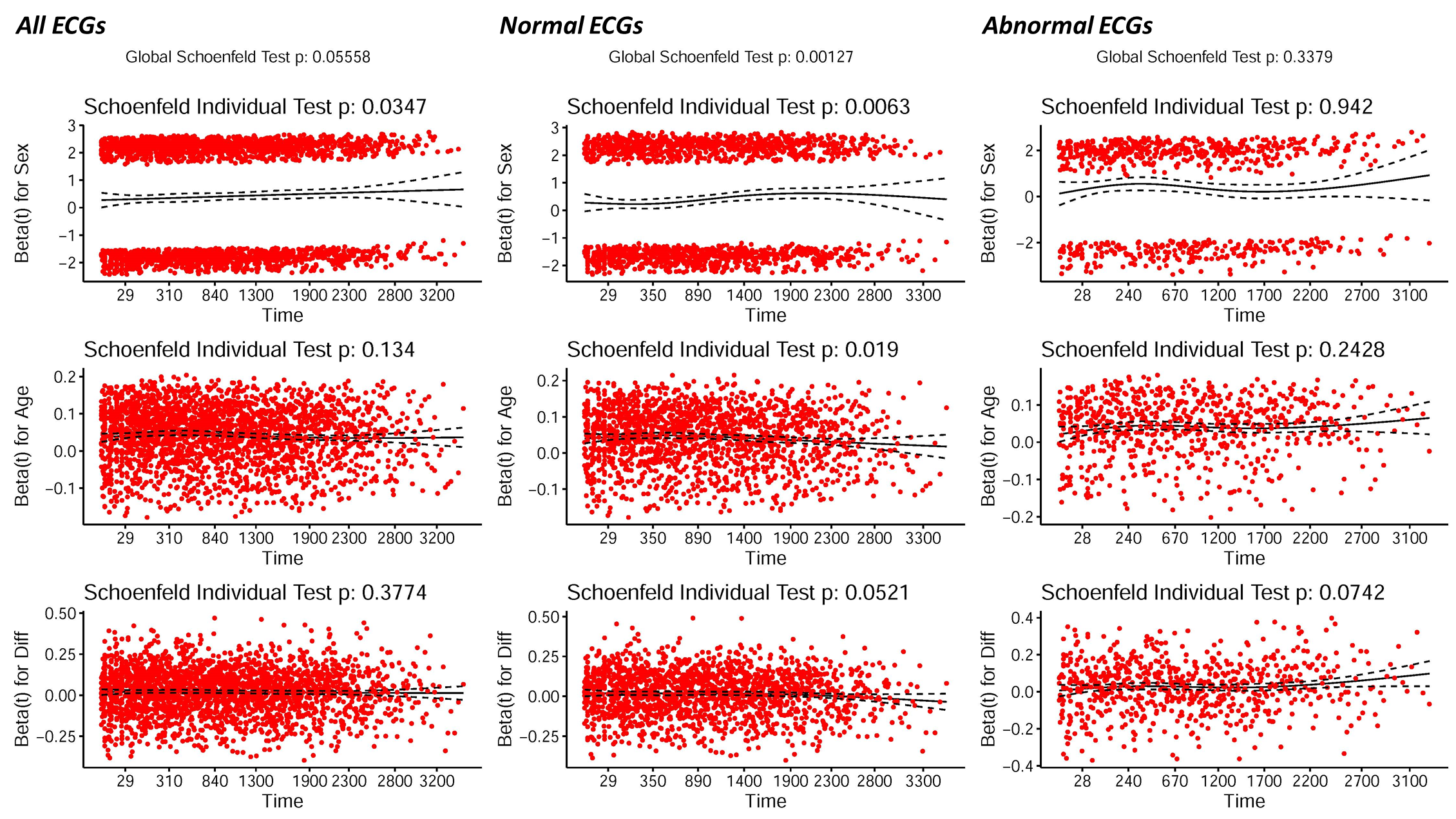


**Supplementary Figure 2-8 | Tests of proportional hazards assumption of Cox models (new-onset CAD).**

We used Global schoenfeld method to test our proportional hazards assumption of Cox models. We have three groups which is All ECGs, normal ECGs and abnormal ECGs. Each category divided into three group which is sex, age and difference (the difference between EC-age and chronologic age).


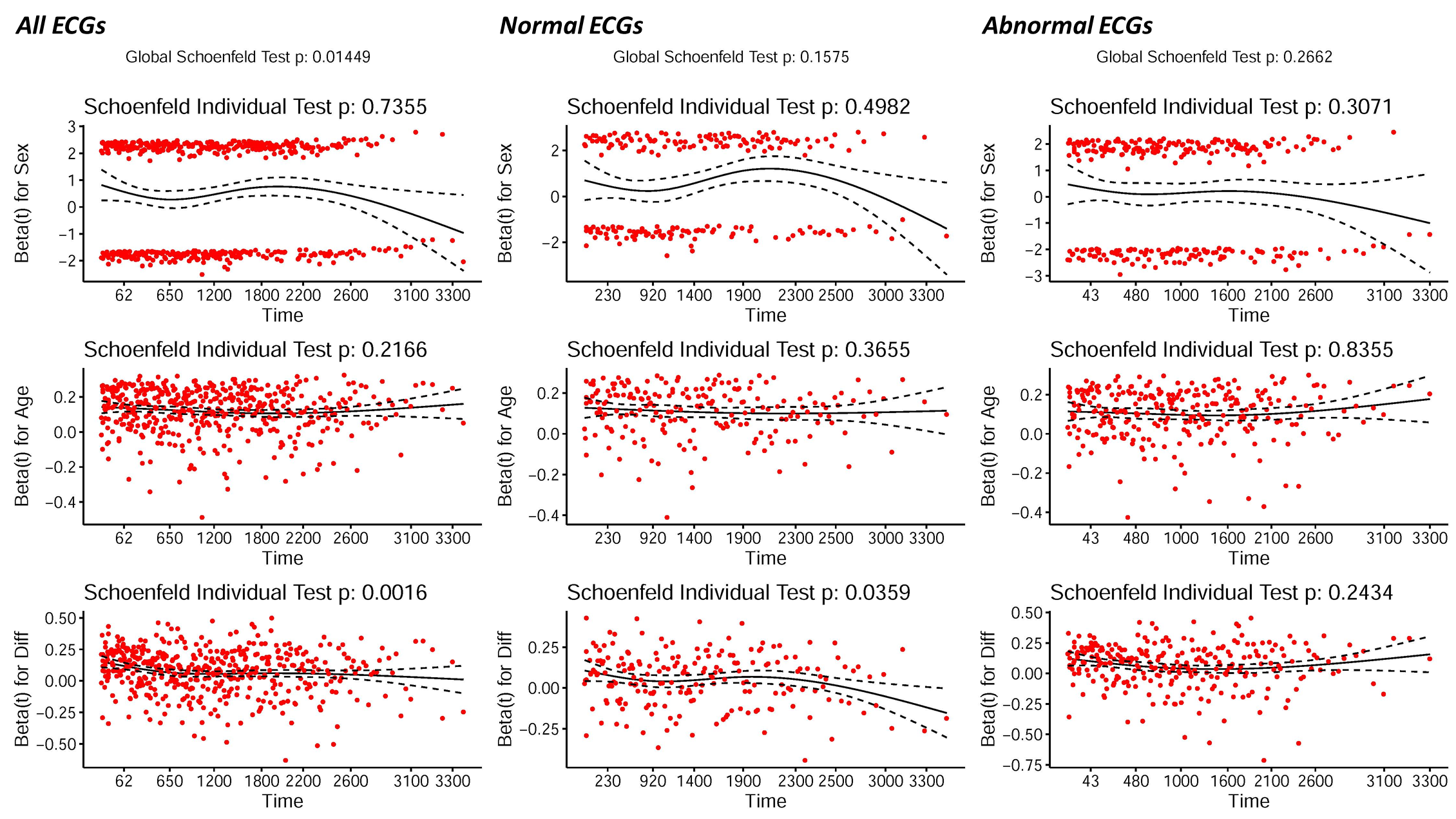


**Supplementary Figure 2-9 | Tests of proportional hazards assumption of Cox models (new-onset AF).**

We used Global schoenfeld method to test our proportional hazards assumption of Cox models. We have three groups which is All ECGs, normal ECGs and abnormal ECGs. Each category divided into three group which is sex, age and difference (the difference between EC-age and chronologic age).


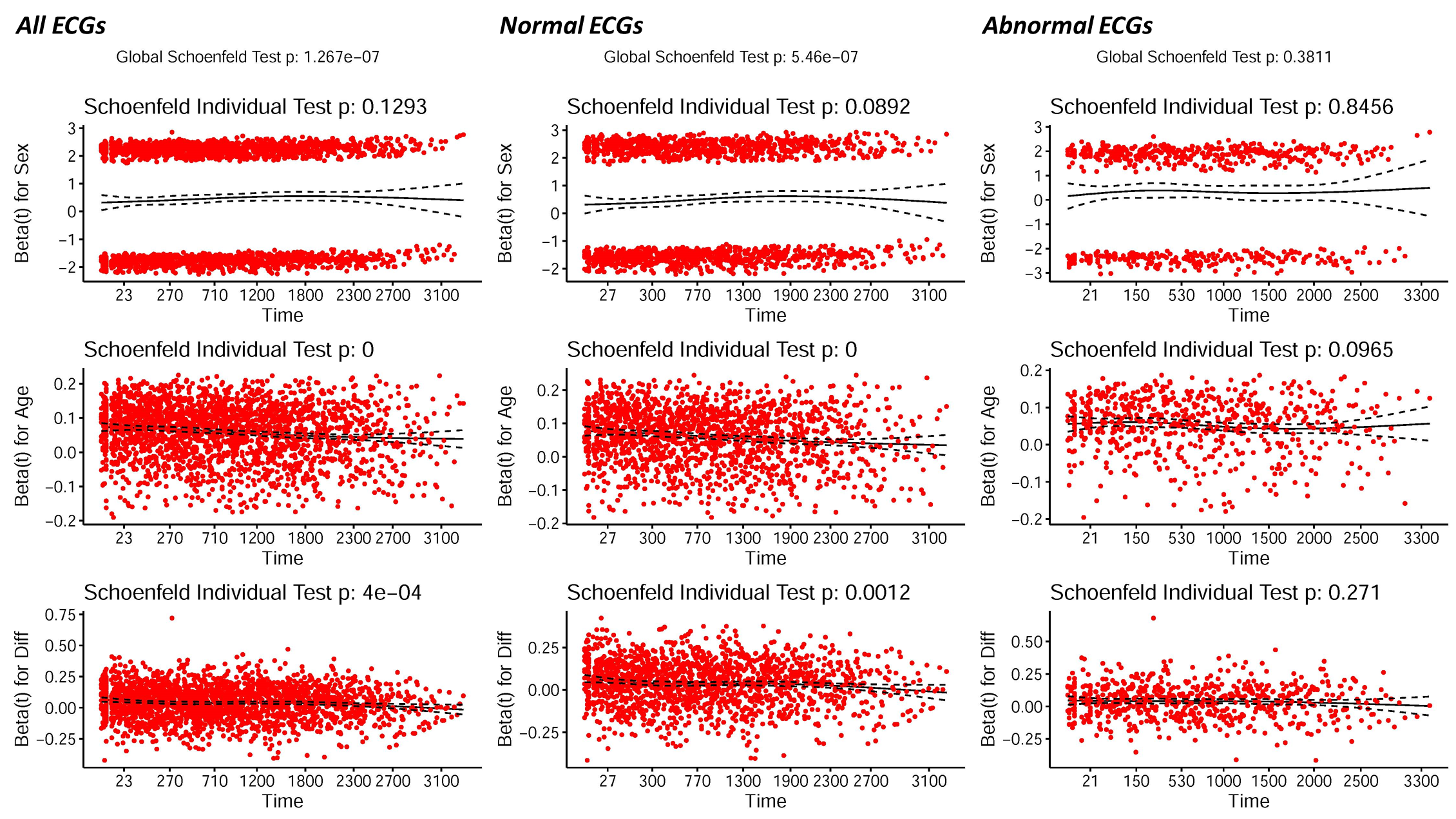


**Supplementary Figure 2-10 | Tests of proportional hazards assumption of Cox models (new-onset HTN).**

We used Global schoenfeld method to test our proportional hazards assumption of Cox models. We have three groups which is All ECGs, normal ECGs and abnormal ECGs. Each category divided into three group which is sex, age and difference (the difference between EC-age and chronologic age).
